# Urea functions as a risk signal driving astrocyte-mediated neuroinflammation following stroke

**DOI:** 10.1186/s12974-025-03620-2

**Published:** 2025-11-24

**Authors:** Yang Han, Tongshuai Zhang, Dandan Wang, Shanshan Yang, Yixiang Jiang, Yao Zhang, Fan Zhu, Haiquan Tao, Liwei Zhao, Xianfeng Li, Haicheng Yang, Jing Lan, Jiachen He, Changyong Tang, Baoxue Yang, Guangzhi Wang, Xuan Li, Guangyou Wang

**Affiliations:** 1 Department of Neurobiology, School of Basic Medical Sciences, Harbin Medical University, Harbin, Heilongjiang 150081 China; 2 Wu Lian De Memorial Hospital, The First Affiliated Hospital of Harbin Medical University, Harbin, Heilongjiang 150001 China; 3 Department of Neurology, The First Affiliated Hospital of Harbin Medical University, Harbin, Heilongjiang 150001 China; 4 Department of Neurobiology, Southern Medical University, Guangzhou, Guangdong 510515 China; 5 Department of Cerebrovascular Disease, Zhuhai People’s Hospital, Zhuhai, Guangdong 519099 China; 6 Department of Neurosurgery, The First Affiliated Hospital of Harbin Medical University, Harbin, Heilongjiang 150001 China; 7 Department of Neurosurgery, The Second Affiliated Hospital of Harbin Medical University, Harbin, Heilongjiang 150086 China; 8 Department of Neurology, The Third Affiliated Hospital of Sun Yat-Sen University, Guangzhou, Guangdong 510630 China; 9 State Key Laboratory of Vascular Homeostasis and Remodeling, Department of Pharmacology, School of Basic Medical Sciences, Peking University, Beijing, 100191 China; 10 Department of Anesthesiology, Harbin Medical University Cancer Hospital, Harbin, Heilongjiang 150081 China; 11 Biotechnology Experimental Teaching Center, Basic Medical College of Harbin Medical University, Harbin, Heilongjiang 150081 China; 12 Key Laboratory of Preservation of Human Genetic Resources and Disease Control in China, Ministry of Education, Harbin Medical University, Harbin, Heilongjiang 150001 China

**Keywords:** Ischemic stroke, Urea, The urea transporter- b (UT-B), Astrocyte, WW domain containing transcription regulator 1 (Wwtr1), Neuroinflammation

## Abstract

Urea, traditionally recognized as the final product of protein metabolism and a regulator of urine concentration, has recently emerged as a potential contributor to central nervous system (CNS) pathologies. However, its role in ischemic stroke remains largely unexplored. In this study, we identify urea as a previously unrecognized risk signal, whose levels rapidly increase in the brain following ischemic stroke. Elevated urea triggers the induction of neurotoxic A1-type astrocytes and promotes neuronal pyroptosis. Mechanistically, this process is mediated by the astrocytic urea transporter UT-B, which facilitates urea influx and initiates a cascade of neuroinflammatory responses. Through quantitative proteomics and gene silencing, we further demonstrate that urea exacerbates DNA damage via activation of the transcriptional coactivator Wwtr1, thereby amplifying the generation of A1 astrocytes. Collectively, our findings reveal a novel pathophysiological role for urea in ischemic brain injury and underscore the critical involvement of astrocytes in modulating neuroinflammation and neuronal death.

**Supplementary Information:**

The online version contains supplementary material available at 10.1186/s12974-025-03620-2.

## Introduction

Stroke remains one of the leading causes of mortality and long-term disability worldwide, imposing a profound socioeconomic burden on patients and their families [1, 2]. Among its subtypes, ischemic stroke is the most prevalent, accounting for approximately 80% of all cases [3, 4]. Within the complex pathophysiology of ischemic stroke, neuroinflammation is one of the central drivers of secondary brain injury, with excessive inflammatory responses strongly correlating with neuronal damage and poorer neurological outcomes [5, 6]. During cerebral ischemia, danger signaling molecules are released from damaged tissues and activate astrocytes, initiating an inflammatory cascade. In turn, activated astrocytes themselves release various neurotoxic mediators, including heat shock proteins and oxidatively damaged DNA, thereby creating a pathological feedback loop that amplifies neuroinflammation and neuronal dysfunction [7–9]. Timely interruption of this feedback loop—by targeting either the risk signals or astrocyte-mediated responses—could be critical for halting secondary neuronal injury and promoting tissue repair. The identification of novel risk signals may thus offer promising targets for therapeutic intervention in ischemic stroke.

Urea, the terminal product of nitrogen metabolism, is synthesized via the urea cycle through the concerted action of five key enzymes [10–12]. This pathway serves as the primary mechanism for detoxifying ammonia in mammals [13]. While traditionally regarded as an inert metabolic waste product, recent studies have implicated elevated brain urea levels in several central nervous system (CNS) disorders. In patients with Alzheimer’s disease and Huntington’s disease, abnormally high concentrations of urea have been observed in brain tissue and are associated with exacerbated neuronal injury [14, 15]. Moreover, pathological accumulation of urea in the hippocampus has been linked to depressive-like behaviors in animal models [16, 17]. Despite these findings, the potential contribution of urea to ischemic stroke pathology remains poorly understood. Due to its high polarity and low lipid solubility, urea cannot readily cross cellular membranes and instead requires facilitation by specific urea transporters encoded by the SLC14 solute carrier family. These transporters are classified into two subtypes: UT-A (*Slc14a2*), primarily expressed in the kidney, and UT-B (*Slc14a1*), which is broadly distributed across mammalian tissues [13, 18–21]. In the CNS, UT-B is predominantly expressed in astrocytes, where it facilitates transmembrane urea transport [22, 23]. However, the role of astrocytic UT-B in CNS pathology, particularly following ischemic injury, remains largely unexplored.

The Hippo signaling pathway, a highly conserved tumor-suppressive cascade, regulates cell proliferation and organ size by modulating the localization and activity of transcriptional coactivators [24–26]. In mammals, phosphorylation of WW-domain-containing transcription regulator 1 (Wwtr1, also known as TAZ) inhibits its nuclear translocation and promotes cytoplasmic degradation, thereby suppressing transcriptional activity [27–29]. Recent evidence indicates that Wwtr1 also plays a central role in inflammation. For instance, Wwtr1 has been shown to intensify inflammatory responses in endothelial cells by modulating hemodynamics [30] and to impair cardiac repair by upregulating interleukin-6 (IL-6) [31]. Furthermore, excessive Wwtr1 activity contributes to oxidative stress and DNA damage, facilitating the progression of liver cancer [32, 33]. These findings suggest a broader role for Wwtr1 in regulating inflammation and genomic instability. Notably, DNA damage not only represents a hallmark of cellular stress but also functions as a alarmin capable of triggering inflammatory responses in the brain [34–36], particularly through astrocytic activation [37]. These insights raise the possibility that Wwtr1 may serve as a molecular link between DNA damage and the neuroinflammatory phenotype of reactive astrocytes.

In this study, we provide the first evidence that urea levels were elevated within infarcted brain tissue of ischemic stroke patients. This finding was subsequently validated in the ischemic brains of mice following permanent middle cerebral artery occlusion (pMCAO). By developing a compound mouse model combining excess cerebral urea and UT-B deficiency, we investigated the contribution of urea to ischemic pathology and its role in driving the generation of neurotoxic A1-type astrocytes. Furthermore, we employed quantitative proteomics and gene silencing approaches to dissect the role of the Wwtr1-DNA damage signaling axis in mediating the phenotypic transformation of reactive astrocytes. Our findings reveal urea as a novel endogenous risk molecule in the ischemic brain and highlight the UT-B transporter as a critical mediator of astrocytic uptake and downstream inflammatory signaling. Inhibition of urea transport significantly ameliorated neuronal injury and improved functional outcomes in ischemic stroke models. These results uncover a previously unrecognized regulatory axis linking urea metabolism to astrocyte-driven neuroinflammation and offer new mechanistic insights and therapeutic avenues for the treatment of ischemic stroke.

## Materials and methods

### Animals, model establishment, and treatment

Male C57BL/6 wild-type mice (20–22 g) were obtained from Liaoning Changsheng Biotechnology Co., Ltd. (Liaoning, China). UT-B knockout (UT-B^−/−^) mice were provided by Dr. Baoxue Yang’s laboratory with C57BL/6 genetic background, generated by targeted deletion of exons 3–6, as previously described [38, 39]. All animals were housed under controlled environmental conditions (12-hour light/dark cycle) with ad libitum access to standard laboratory chow and water.

All animal procedures were conducted in accordance with the Guidelines for the Care and Use of Laboratory Animals issued by the China National Institute of Health and adhered to the ARRIVE guidelines. The experimental protocol was reviewed and approved by the Institutional Animal Care and Use Committee of Harbin Medical University (Ethics Approval No.: [HMUIRB2025006]).

### Alzet-osmotic pump administration

Urea (Merck, Germany), the UT-B inhibitor UT-B-IN-1 (MCE, USA) and the Wwtr1 inhibitor-EMT inhibitor-1 (MCE, USA) were dissolved in 0.9% sterile saline. The prepared solutions—sterile saline (vehicle), urea, UT-B-IN-1 or EMT inhibitor-1—were loaded into Alzet osmotic pumps (model 1003D, Alzet). Each pump was surgically implanted subcutaneously in the dorsal region of the mice, and connected to a brain infusion catheter. The catheter was stereotactically positioned near the target area corresponding to the permanent middle cerebral artery occlusion (pMCAO) model and secured using dental cement suitable for mice.

Following implantation, the skin was sutured. After 3 days of continuous infusion, both the pump and catheter were removed, and the pMCAO model was established as described in subsequent sections.

### pMCAO model establishment

Mice were anesthetized with 2% pentobarbital sodium administered intraperitoneally at a dose of 5 mL/kg. Under a surgical microscope, a midline incision was made in the neck to expose and ligate the common carotid artery. Subsequently, a second incision was made from the left eye to the anterior region of the skull. The skull was carefully drilled to expose the left middle cerebral artery (MCA), which was then occluded using an electrocoagulation pen. All procedures were performed with precision and expedience to minimize bleeding and reduce surgical trauma.

Following successful occlusion of the MCA, the ligation of the common carotid artery was released, and all surgical incisions were sutured.

### Clinical sample collection and use

Brain tissue samples used in this study included

1. Cerebral infarction group: Ischemic brain tissue from stroke patients undergoing surgery post-onset.
2. Control group: Non-functional brain regions resected during decompressive surgery in patients with temporal tumor, cerebral aneurysm or carotid artery aneurysm.

All procedures were performed with obtained informed consent from participants, and all specimens were pathologically confirmed and ethically approved by the Second Affiliated Hospital of Harbin Medical University (Ethics Approval No.: [YJSKY2023-498]).

### Behavioral testing

Three assays were used in this work: Open field test (OFT), Grip strength test (GST) and Rotarod test (RRT). The open field test was conducted to assess spontaneous motor activity and exploratory behavior following surgery. Mice were placed in an open-field arena (50 × 50 × 30 cm³), with four animals tested simultaneously. The total distance traveled and average movement speed were recorded. After each session, the arena floor was cleaned with 75% ethanol to eliminate olfactory cues and prevent interference with subsequent trials. The grip strength test was employed to evaluate forelimb muscle strength as an indicator of overall neuromuscular function. Each mouse was placed on a grip strength meter, and its tail was gently pulled backward to prompt the animal to grasp the probe with its forelimbs. The peak force reading displayed on the meter was recorded for analysis. To assess motor coordination and balance, mice were tested using an accelerating rotarod apparatus. Prior to the test, mice underwent a 5-minute training session at a constant speed. For the formal test, the rod was set to accelerate gradually, reaching a maximum speed of 40 revolutions per minute. The latency to fall—defined as the time elapsed from placement on the rod to the moment the mouse fell—was recorded.

### TTC staining

2,3,5-Triphenyltetrazolium chloride (TTC) staining is a commonly used method to assess cerebral infarct volume in ischemic stroke models. TTC is a colorless compound that is enzymatically reduced by active mitochondrial dehydrogenases in viable cells to form a red formazan dye. Consequently, living brain tissue stains red, while infarcted tissue, remains unstained or appears white.

After cervical dislocation of mice, the brain tissues were rapidly extracted and were precisely sectioned into uniformly thick 1–2 mm coronal slices and completely immersed in 2% TTC staining solution, followed by incubation at 37℃ in the dark for 30 min. Upon completion of staining, the reaction was immediately terminated with 4% paraformaldehyde solution and fixed overnight at 4℃. On the following day, brain sections were imaged, and the total area as well as the white infarct area were precisely measured using ImageJ software to calculate the infarct volume percentage for assessing ischemic damage.

### BBB permeability assay

Blood–brain barrier (BBB) permeability was assessed using the Evans Blue dye extravasation method. Evans Blue (Merck, Germany) was administered via the tail vein 2 h prior to euthanasia. Following perfusion and brain extraction, tissues were homogenized and centrifuged to obtain the supernatant. The degree of BBB disruption was quantified by measuring the absorbance of the supernatant at 620 nm using a microplate reader.

### Hematoxylin-eosin staining (H&E staining)

Brain tissues were fixed in 4% paraformaldehyde and processed into frozen sections. Hematoxylin–eosin (H&E) staining was performed to assess neuronal morphology and the extent of inflammatory cell infiltration in the brain tissue.

### Cell culture

Primary astrocytes were isolated from neonatal mice within 48 h of birth. Following surface disinfection with 75% ethanol, cerebral cortices were aseptically dissected and transferred into F12 medium. The tissues were mechanically dissociated into single-cell suspensions and filtered through a 40-µm cell strainer. The cell suspension was centrifuged at 1600 rpm for 5 min, the supernatant was discarded, and the pellet was resuspended in astrocyte culture medium. Cells were seeded into poly-D-lysine-coated culture flasks and incubated overnight. The culture medium was replaced every 3 days. After 9–10 days of culture, the astrocytes were ready for experimental use. Immunofluorescence staining confirmed astrocyte purity exceeding 90%.

Primary cortical neurons were isolated from neonatal mice within 24 h of birth. Following the removal of meninges, cerebral cortex tissues were dissected and placed in F12 medium. The tissue was gently triturated using a pipette, and the resulting suspension was filtered through a 40-µm cell strainer. After centrifugation at 1600 rpm for 5 min, the cell pellet was resuspended and seeded into poly-D-lysine-coated flasks. After 4–6 h, the medium was replaced with F12 supplemented with B27. Thereafter, the medium was changed every 2 days. Cells were used for experiments once they reached 80–90% confluency. All cell cultures were maintained in a humidified incubator at 37℃ with 5% CO₂.

### The Oxygen-glucose deprivation (OGD) assay

To simulate the ischemic environment in vitro, the oxygen–glucose deprivation (OGD) assay was performed. The original culture medium was replaced with glucose-free medium, and the cells were incubated at 37℃ in a hypoxic chamber containing 95% nitrogen (N₂) and 5% carbon dioxide (CO₂).

### Small interfering RNA (siRNA) transfection

Primary astrocytes were seeded in 6-well plates and transfected with siRNA once they reached 70–80% confluency. Transfection was performed using Lipofectamine™ 3000 (Thermo Fisher Scientific, USA) following the manufacturer’s protocol. Cells were incubated with the transfection complex for 48–72 h prior to downstream analysis. The siRNA sequences targeting *Wwtr1* were as follows: Sense (S): 5’- CCACUGGCCAGAGAUACUU- 3’; Antisense (AS): 5’- AAGUAUCUCUGGCCAGUGG-3’.

### Cell viability assay

Primary astrocytes and neurons were seeded into 96-well plates and allowed to adhere fully before initiating experimental treatments. Following treatment, 10 µL of Cell Counting Kit-8 (CCK-8, TargetMol, USA) reagent was added to each well. The plates were incubated at 37 °C for 2.5 h, after which absorbance was measured at 450 nm using a microplate reader. Cell viability was expressed as a percentage relative to control wells.

### Western blot

Brain tissue and cultured cell samples were lysed in RIPA buffer, and total protein was extracted. Protein concentrations were determined using a BCA assay kit (Beyotime, China), and adjusted samples were separated by SDS-PAGE, followed by transfer to polyvinylidene difluoride (PVDF) membranes. Membranes were blocked with 5% skim milk for 1–2 h at room temperature and incubated overnight at 4 °C with primary antibodies. The following targets were assessed: urea cycle enzymes (ASS1 (1:600, Cell Signaling Technology, USA), ASL (1:500, GeneTex, USA), ARG1 (1:500, ABclonal, China), OTC (1:500, Abcam, UK), CPS1 (1:500, Abcam, UK)), the urea transporter UT-B (1:400, Thermo Fisher, USA), astrocyte and inflammatory markers (C3 (1:400, Thermo Fisher, USA), Wwtr1 (1:500, Cell Signaling Technology, USA), TNF-α (1:500, Abcam, UK), IL-1β (1:500, Cell Signaling Technology, USA)), pyroptosis-related proteins (NLRP3 (1:800, abcam, UK), GSDMD (1:800, abcam, UK), GSDMD-N (1:800, ImmunoWay, China), IL-18 (1:500, Cell Signaling Technology, USA), Caspase-1/Active caspase-1 (1:500, Cell Signaling Technology, USA)), apoptosis-related proteins (Bax (1:800, Cell Signaling Technology, USA), Bcl2 (1:800, ImmunoWay, China)), and β-actin (1:1500, ZSGB-BIO, China) at 4℃ overnight. Subsequently, was used as the loading control. Following incubation with primary antibodies, the membranes were washed and incubated with horseradish peroxidase (HRP)-conjugated secondary antibodies at room temperature for 1–2 h. Protein signals were subsequently detected using an enhanced chemiluminescence (ECL) reagent.

### Total RNA extraction and quantitative PCR

Total RNA was extracted from brain tissues or cultured cells using TRIzol reagent (TaKaRa, Japan), following the manufacturer’s protocol. The isolated RNA was then reverse-transcribed into complementary DNA (cDNA). Real-time quantitative PCR (qPCR) was conducted using SYBR Green reagent (TransGen Biotech, China) on an ABI qPCR system. Gene expression levels were normalized to the internal control gene *18 S rRNA* and quantified using the 2^-ΔΔCt method.

### Immunofluorescence staining

Clinical brain tissue samples were processed for paraffin embedding and sectioned at 4 μm. Or, alternatively mice brain tissues were embedded in optimal cutting temperature (OCT) compound and coronally sectioned at 10 μm thickness. For in vitro studies, primary astrocytes were cultured on cell culture slides. Both tissue sections and cell slides were fixed with either cold acetone or 4% paraformaldehyde for 10 min. Samples were then blocked with 5% bovine serum albumin containing 0.3% Triton X-100 for 1.5 h at room temperature. Subsequently, they were incubated overnight at 4 °C with the following primary antibodies: anti-GFAP (1:500, Abcam, UK), anti-C3 (1:200), anti-UT-B (1:200), anti-Wwtr1 (1:200), and anti-γ-H2AX (1:200, Abcam, UK). On the following day, samples were incubated with Alexa Fluor 488- or 594-conjugated secondary antibodies (1:500, Jackson ImmunoResearch, USA) for 1 h at room temperature in the dark. Nuclear counterstaining was performed using DAPI (Beyotime, China). Finally, the sections were observed and photographed using a confocal microscope (Zeiss, Germany). Additionally, Sholl analysis of astrocytes was performed using Fiji-ImageJ software.

### Urea concentration detection

Serum samples were collected from experimental mice and left at room temperature for 1 h, then centrifuged to isolate the supernatant for analysis. For brain tissue, mice tissues from specified brain regions and human brain tissues were homogenized and centrifuged. The resulting supernatants were used for urea quantification. After various experimental treatments, the culture medium from primary astrocyte cultures was collected and centrifuged at low speed to obtain cell-free supernatants. In parallel, cells were lysed in 100 µL of urea detection buffer, and the lysates were centrifuged to obtain intracellular content for analysis. All supernatants were processed using a commercial urea detection kit (Abcam, UK). Following the manufacturer’s instructions, the reagent was added to the samples, incubated to allow full reaction, and optical density (OD) was measured at 570 nm using a microplate reader. Urea concentrations were calculated based on standard curves.

### Proteomics

Primary astrocytes were divided into four experimental groups: Control, Urea, OGD (oxygen-glucose deprivation for 4 h), and Urea + OGD (4 h). After completion of the respective treatments, cell lysates were collected, and protein supernatants were prepared for subsequent quantitative proteomic analysis.

### Enzyme-linked immunosorbent assays (ELISAs)

The concentrations of TNF-α and IL-1β in the culture supernatants of primary astrocytes were quantified using commercially available ELISA kits. TNF-α levels were measured using the NEOBIOSCIENCE EMC102a.96 kit, and IL-1β levels were assessed using the NEOBIOSCIENCE EMC001b.96 kit, following the manufacturers’ protocols.

### Statistical analysis

All experiments were performed with a minimum of three biological replicates. Data are presented as mean ± standard error of the mean (SEM). Statistical comparisons between two groups were analyzed using the Student’s *t* test or one-way analysis of variance (ANOVA) were employed for data analysis involving more than two groups, as appropriate. A p-value of less than 0.05 was considered statistically significant (*P < 0.05; **P < 0.01; ***P < 0.001). GraphPad Prism 9 (GraphPad Software, CA) was used for these statistical analyses.

## Results

### Elevated Urea levels in the ischemic hemisphere following cerebral ischemic stroke

Recent studies have increasingly implicated urea in CNS pathologies, particularly neurodegenerative diseases. However, its pathological role and dynamic regulation during ischemic stroke remain largely unexplored. To address this knowledge gap, we first investigated the association between cerebral ischemia and changes in brain urea levels.

**Fig. 1:**
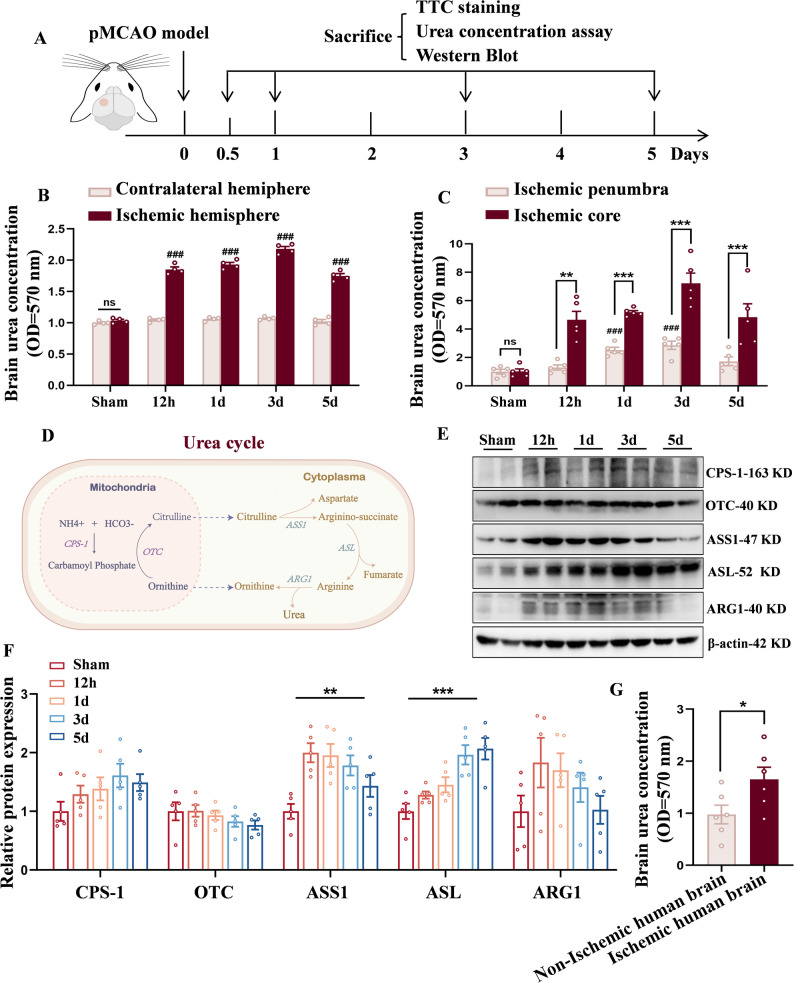
Elevated urea levels in the ischemic hemisphere following cerebral ischemic stroke. **A** The permanent middle cerebral artery occlusion (pMCAO) model was established to assess urea concentrations and infarct size in mouse brains. **B** Urea levels in the ischemic hemisphere increased with prolonged ischemia, while levels in the contralateral hemisphere remained unchanged. *n* = 4 per group. Two-Way ANOVA, Mean ± SEM. **C** Urea concentration was significantly higher in the ischemic core than in the penumbra. *n* = 5 per group. Two-Way ANOVA, Mean ± SEM. **D** Schematic diagram of the classical mammalian urea cycle. **E**, **F** Protein expression of urea cycle enzymes ASS1 and ASL were upregulated following pMCAO. *n* = 5 per group. One-Way ANOVA, Mean ± SEM. **G** Elevated ureal levels in infarcted brain tissues compared to unaffected regions in patients with ischemic stroke. *n* = 6 per group. Student’s *t* tests. Mean ± SEM. *P < 0.05, **P < 0.01, ***P < 0.001, *ns=* not significant, # compared with Sham group

Using male C57BL/6 mice, we established a permanent middle cerebral artery occlusion (pMCAO) model (Fig. 1A). TTC staining revealed a time-dependent expansion of the infarcted area with prolonged ischemia (Fig. S1A, S1B). Correspondingly, biochemical assays revealed a significant increase in urea concentration in the ischemic hemisphere of the ischemic group compared to the sham group, while urea levels in the contralateral hemisphere and serum remained unchanged (Fig. 1B; Fig. S1C). Subsequent regional distribution analysis showed markedly higher urea concentrations in the ischemic core compared to the surrounding penumbra (Fig. 1C), suggesting that the elevated urea predominantly originates from the core, likely attributable to cellular necrosis and metabolic disruption [40, 41]. To explore the potential source of increased urea, we performed Western Blot analysis to assess the expression of key urea cycle enzymes. Results revealed upregulation of multiple enzymes involved in urea biosynthesis within the ischemic hemisphere (Fig. 1D and F), indicating an enhancement of urea cycle activity under ischemic conditions.

Meantime, we performed a retrospective analysis of infarcted brain tissue resected from stroke patients. Results demonstrated elevated urea levels in the infarcted regions compared to controls (Fig. 1G), consistent with our previous experimental data. Collectively, these findings provide novel experimental evidence that cerebral ischemia leads to region-specific accumulation of urea, driven by enhanced local metabolism and cellular damage. This establishes a potential pathophysiological link between urea and ischemic stroke progression.

### Elevated brain urea levels exacerbate ischemic brain injury

**Fig. 2:**
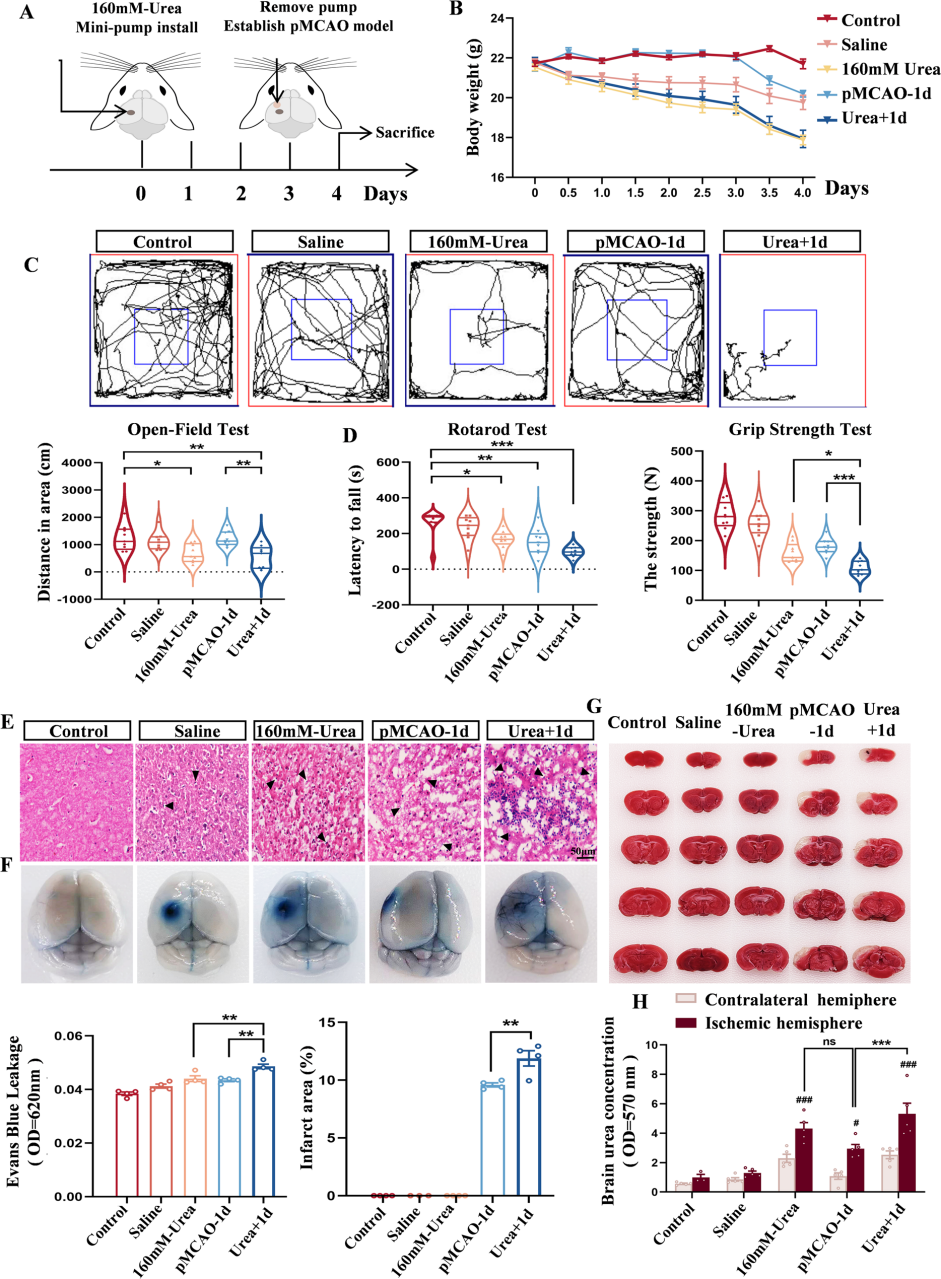
Elevated brain urea levels exacerbate ischemic brain injury. **A** Experimental setup of the Minipump (160 mM urea) + pMCAO (1 day) model. **B** Body weight changes recorded across treatment groups. *n* = 9 per group. One-Way ANOVA, Mean ± SEM. **C** Open field test assessing. locomotor activity and total distance traveled. *n* = 9 per group. One-Way ANOVA, Mean ± SEM. **D** Rotarod test evaluating limb coordination and balance, and grip strength test measuring forelimb strength. *n* = 9 per group. One-Way ANOVA, Mean ± SEM. **E** H&E-stained brain tissue section (200× magnification) revealed significant disruption of the histological architecture in the experimental group, characterized by loosened tissue structure, widened intercellular spaces, disorganized cellular arrangement, and pyknotic nuclei (▲). **F** Evans Blue dye accumulation indicating blood–brain barrier (BBB) permeability; the Urea + 1 d group showed the most severe BBB disruption. *n* = 4 per group. One-Way ANOVA, Mean ± SEM. **G** TTC staining revealed the largest infarct area in the Urea + 1 d group. *n* = 4 per group. One-Way ANOVA, Mean ± SEM. **H** Quantitative analysis of urea concentration in bilateral hemispheres across all groups. *n* = 5 per group. Two-Way ANOVA, Mean ± SEM. *P < 0.05, **P < 0.01, ***P < 0.001. *ns =* not significant, # compared with Control group

Dysregulated urea metabolism has been implicated in several neurological disorders. For instance, Yeon Ha Ju et al. reported that in Alzheimer’s disease, astrocytes upregulate the urea cycle to detoxify amyloid-β (Aβ) deposits but simultaneously produce excess urea, ammonia, and reactive oxygen species, contributing to neuronal damage [14]. To explore whether urea exerts similar deleterious effects in the context of cerebral ischemia, we established a model of urea overaccumulation in the ischemic brain. Given urea’s high osmotic activity [42], which could induce cellular dehydration or necrosis upon direct injection, we utilized Alzet osmotic pumps to achieve controlled, sustained intracerebral delivery. Based on the metabolic kinetics of urea in the mouse brain and its effects on survival, we established a Minipump (160 mM urea) + pMCAO (1-day) model (Fig. 2A).

Male mice were randomly assigned to five groups: blank control, vehicle control (0.9% saline), 160 mM urea only, pMCAO-1d, and Urea + 1d. Mice in the Urea + 1 d group displayed marked lethargy, decreased food and water intake, and significant body weight loss throughout the experiment (Fig. 2B). Behavioral assessments conducted prior to sampling revealed pronounced neurological deficits in the experimental groups. Open field testing indicated normal activity in the control and saline groups, whereas all other groups showed reduced exploratory behavior, most notably in the Urea + 1 d group (Fig. 2C). Similarly, performance on the rotarod and grip strength tests was significantly impaired by both urea administration and ischemia, with the Urea + 1 d group exhibiting the most severe motor dysfunction (Fig. 2D).

Histopathological analysis using H&E staining further confirmed these behavioral findings. Control animals displayed intact cortical architecture with orderly neuronal arrangement and preserved nuclear morphology. In contrast, the urea + 1 d group exhibited significant histopathological damage, characterized by: disorganized cellular arrangement, widened intercellular spaces, and prominent nuclear pyknosis or loss. Indicates extensive neuronal death. Moreover, this group displayed enhanced inflammatory cell infiltration (Fig. 2E). Evaluation of blood-brain barrier (BBB) integrity via Evans Blue extravasation demonstrated that both urea administration and ischemia increased BBB permeability. The urea + 1 d group exhibited the most severe compromise, as quantified by heightened Evans Blue accumulation in cerebral tissue (Fig. 2F). TTC staining revealed that urea exacerbated infarct size: mice in the Urea + 1 d group had significantly larger infarcts than those in the urea-only or pMCAO-only groups, indicating synergistic exacerbation of tissue injury (Fig. 2G).

Quantification of brain urea levels showed elevated concentrations in the ischemic hemispheres of all groups subjected to urea infusion or ischemia. The Urea + 1 d group had higher urea levels than either the 160 mM urea or pMCAO-1d groups alone (Fig. 2H). Notably, urea concentrations in the 160 mM urea and pMCAO-1d groups were comparable, suggesting that exogenous infusion mimics endogenous accumulation after stroke. These findings demonstrate that elevated cerebral urea, whether from ischemic pathology or exogenous infusion, is sufficient to induce neuronal injury and exacerbate stroke-related outcomes. Importantly, the observed effects are not attributable to osmotic pump implantation or vehicle administration. Moreover, the positive correlation between post-ischemic urea levels and the extent of brain damage highlights urea as a potential pathogenic factor in ischemic stroke, offering a novel direction for mechanistic and therapeutic research in cerebrovascular disease.

### Urea induces the generation of neurotoxic astrocytes in ischemic brain tissue, leading to reduced neuronal activity

**Fig. 3:**
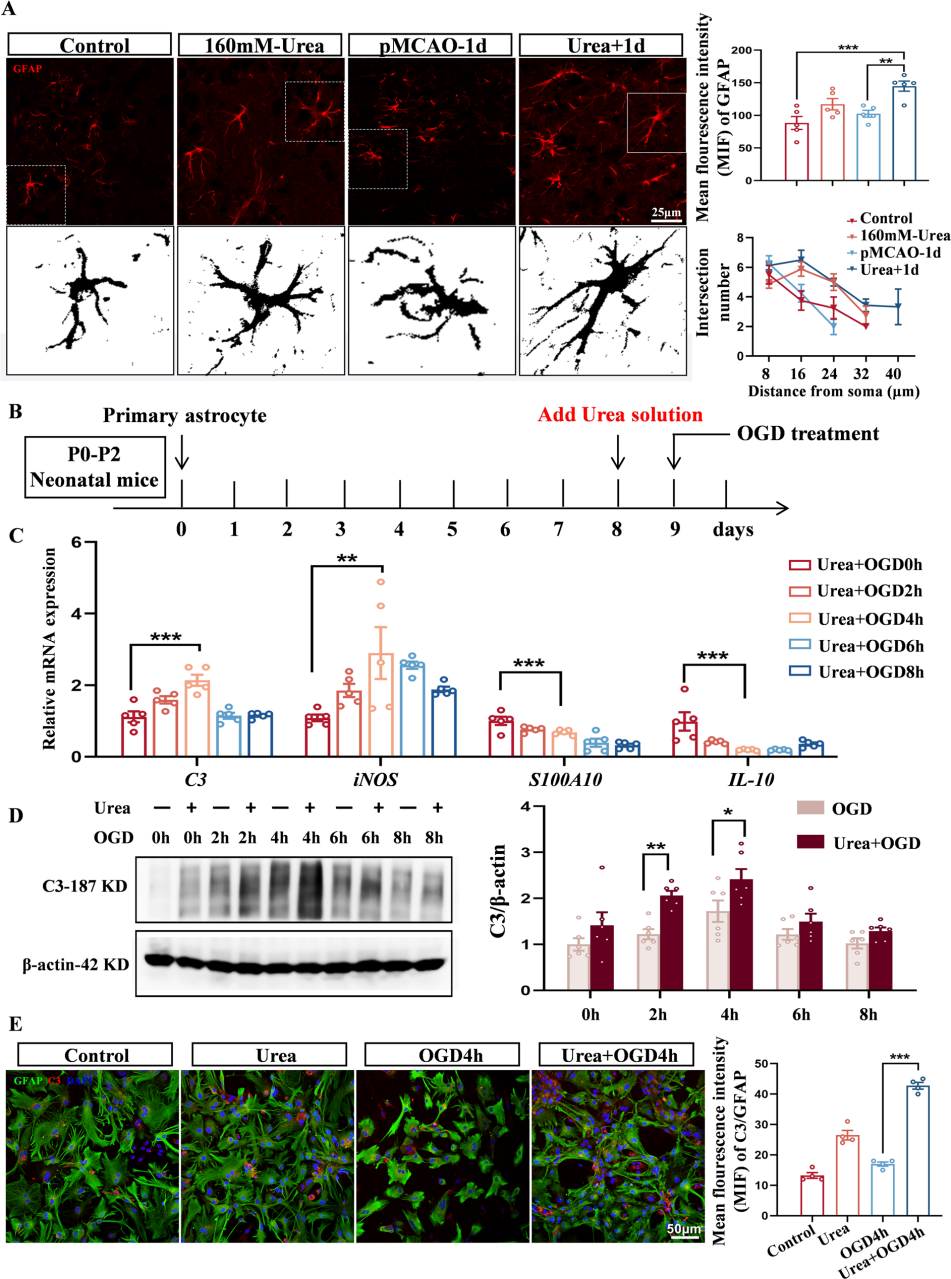
Urea induces the generation of neurotoxic astrocytes in ischemic brain tissue. **A** Representative images of GFAP^+^ astrocyte and quantitative analysis of process branching determined by Sholl analysis. *n* = 5 per group. One-Way ANOVA, Mean ± SEM. **B** In vitro co-treatment of primary astrocytes with 160 mM urea and oxygen-glucose deprivation (OGD). **C** After 24-hour urea treatment, OGD further increased expression of A1-associated genes (*C3*,* iNOS*) and decreased A2-associated genes (*S100A10*,* IL-10*). *n* = 4 per group. One-Way ANOVA, Mean ± SEM. **D** Western Blot comparison of C3 protein levels between OGD and Urea + OGD astrocyte groups. *n* = 6 per group. Two-Way ANOVA, Mean ± SEM. **E** Immunofluorescence co-staining of C3 (red) and GFAP (green) in astrocytes across different groups. Scale bar = 50 μm. *n* = 4 per group. One-Way ANOVA, Mean ± SEM. *P < 0.05, **P < 0.01, ***P < 0.001

To further elucidate the mechanisms by which urea exacerbates ischemic brain injury, we investigated the role of reactive astrocytes. Following CNS injury, astrocytes undergo extensive morphological and functional remodeling and can adopt distinct phenotypes. Based on transcriptomic and functional profiles, reactive astrocytes are broadly classified into neurotoxic A1 and neuroprotective A2 subtypes. The excessive activation of A1 astrocytes is a hallmark of heightened neuroinflammation and neuronal damage in multiple disease models [43–46]. We hypothesized that elevated urea levels promote the polarization of astrocytes toward the A1 phenotype, thereby amplifying the inflammatory response after ischemic stroke. To test this, we examined the molecular profiles of ischemic brain tissues in the presence or absence of urea overload. Immunofluorescence staining revealed significant morphological changes in astrocytes within the ischemic penumbra of Urea + 1 d group mice, including marked upregulated expression of glial fibrillary acidic protein (GFAP), as well as elongated and more branched processes (Fig. 3A). Furthermore, these activated astrocytes exhibited a characteristic shift in phenotype, with increased expression of A1-specific markers and decreased expression of A2 markers (Fig. S1D, S1E). Meanwhile, expression of chemokines *CCL2* and *CXCL10* was markedly increased (Fig. S1F), suggesting enhanced recruitment of peripheral immune cells—consistent with their established role in mediating leukocyte infiltration across the blood–brain barrier [47–49].

To directly assess the effect of urea on astrocyte polarization, we conducted in vitro experiments using primary astrocytes (Fig. 3B). Cell viability assays and urea concentration measurements confirmed that treatment with 160 mM urea enhanced astrocyte activity independently of osmotic stress (Fig. S2A, S2B), and that astrocytes are capable of actively absorbing exogenous urea (Fig. S2C). Following 24-hours exposure to urea under OGD conditions, expression of A1 markers (*C3 and iNOS*) increased significantly, whereas A2 markers (*S100A10 and IL-10*) were suppressed (Fig. 3C). Notably, C3 protein levels were significantly higher in the Urea + OGD group compared to OGD alone (Fig. 3D and E), consistent with in vivo observations and supporting urea’s role in driving A1 astrocyte polarization. To compare the inflammatory potency of urea with a known stimulus, we assessed cytokine expression in astrocytes treated with Urea + OGD and those exposed to lipopolysaccharide (LPS, 1 µg/mL) [50, 51]. qPCR analysis revealed comparable levels of pro-inflammatory cytokines expression between the two conditions (Fig. S2D, S2E), indicating that urea elicits a strong pro-inflammatory response in astrocytes, on par with classical immune stimuli.

**Fig. 4:**
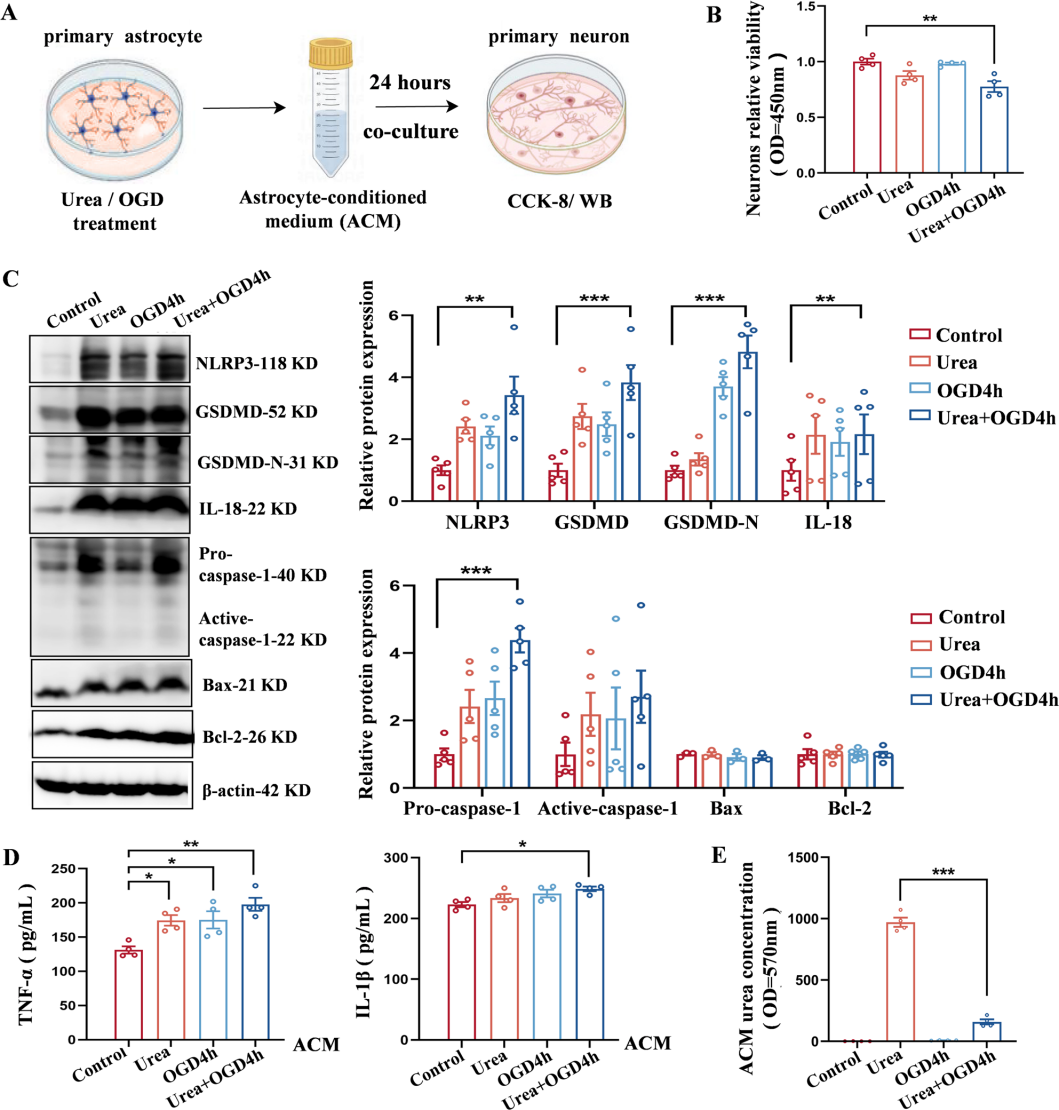
Urea-induced production of neurotoxic astrocytes leads to neuronal damage. **A** Astrocyte–neuron co-culture model: astrocyte-conditioned medium (ACM) from urea- and OGD-treated astrocytes was applied to primary neurons for 24 h. **B** Neuronal viability assessed by CCK-8 assay. *n* = 4 per group. One-Way ANOVA, Mean ± SEM. **C** Expression of pyroptosis-related proteins in neurons exposed to different ACM treatments. *n* = 5 per group. One-Way ANOVA, Mean ± SEM. **D** ELISA quantification of TNF-α and IL-1β levels in ACM. *n* = 4 per group. One-Way ANOVA, Mean ± SEM. **E** Urea concentration measured across different ACM groups. *n* = 4 per group. One-Way ANOVA, Mean ± SEM. *P < 0.05, **P < 0.01, ***P < 0.001

Given that A1 astrocytes are known to induce neuronal death via oxidative stress, excitotoxicity, and neuroinflammation [52–54], we next evaluated the impact of urea-induced astrocyte activation on neuronal viability. To this end, we established an astrocyte–neuron co-culture system using astrocyte-conditioned medium (ACM) from various treatment groups (Fig. 4A). After 24 h of exposure, CCK-8 assays revealed the lowest neuronal viability in the Urea + OGD4h group (Fig. 4B). Although levels of classical apoptotic proteins Bax and Bcl-2 remained unchanged, pyroptosis-related proteins—including NLRP3, GSDMD, GSDMD-N, Caspase-1, and IL-18—were markedly upregulated in neurons treated with Urea + OGD ACM (Fig. 4C).

Finally, we analyzed urea concentrations and cytokine levels in the ACM. Notably, although urea levels were lower in the Urea + OGD4h group compared to the Urea-only group, levels of TNF-α and IL-1β were higher (Fig. 4D and E). These findings indicate that the deleterious effects on neurons are not due to residual urea itself, but rather to the inflammatory mediators secreted by urea-polarized A1 astrocytes. Together, these data establish a mechanistic link between elevated brain urea, astrocyte polarization toward a neurotoxic phenotype, and neuronal pyroptosis following ischemic stroke.

#### Functional disruption of UT-B protects against urea-mediated brain injury

Urea transporters, encoded by members of the solute carrier (SLC) family, are essential for mediating urea transmembrane transport. In the CNS, UT-B (*Slc14a1*) plays a predominant role in maintaining brain urea homeostasis, whereas UT-A (*Slc14a2)* is primarily restricted to renal tissues. To investigate this, we examined the expression of UT-A and UT-B in brain tissues and primary astrocytes under various conditions. Both in vivo and in vitro experiments demonstrated that pMCAO/OGD and urea treatment significantly upregulated UT-B expression, while UT-A levels remained unchanged (Fig. S3A-S3F), justifying UT-B as the primary focus of functional investigation. Furthermore, immunofluorescence staining revealed upregulated UT-B protein expression in astrocytes within the infarcted regions of stroke patients (Fig. S3G). To determine the contribution of UT-B to urea-induced pathology, we utilized UT-B knockout (UT-B^−/−^) mice and confirmed complete loss of UT-B protein expression in brain tissue (Fig. S4A).

**Fig. 5:**
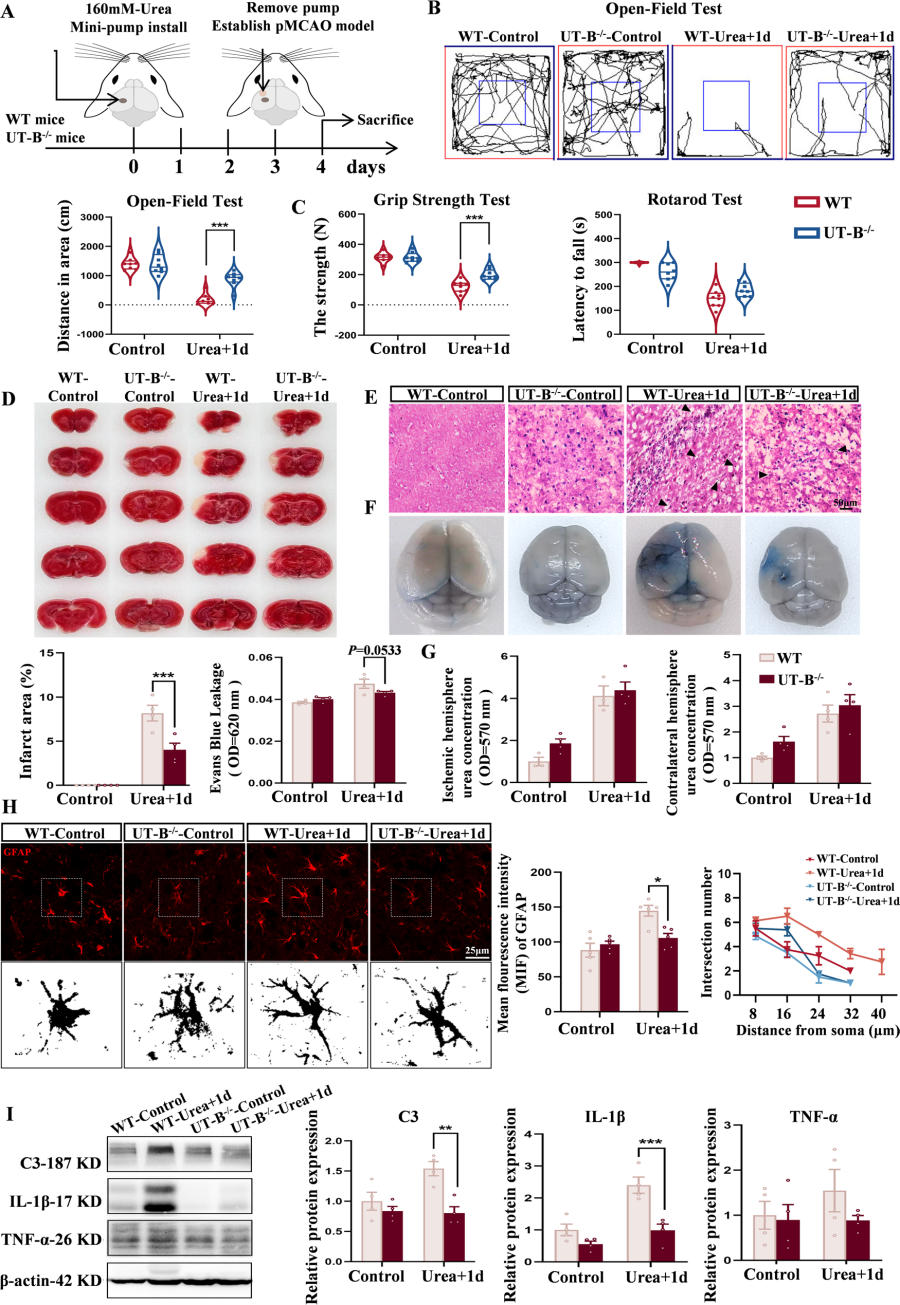
UT-B^−/−^ mice are resistant to urea-mediated brain damage. **A** Experimental setup of the Minipump (160 mM urea) + pMCAO (1 day) model. **B** Open field test assessing locomotor activity and total distance traveled in WT and UT-B^−/−^ mice. *n* = 8 per group. Two-Way ANOVA, Mean ± SEM. **C** Rotarod and grip strength tests evaluating motor coordination and forelimb strength. *n* = 8 per group. Two-Way ANOVA, Mean ± SEM. **D** TTC staining showing reduced infarct size in UT-B^−/−^ brains. *n* = 4 per group. Two-Way ANOVA, Mean ± SEM. **E** H&E staining assessing cellular morphology and histological integrity (200× magnification). **F** Evans Blue staining indicating preserved BBB integrity in UT-B^−/−^ mice. *n* = 4 per group. Two-Way ANOVA, Mean ± SEM. **G** Urea concentration in ischemic and contralateral hemisphere across four experimental groups. *n* = 4 per group. Two-Way ANOVA, Mean ± SEM. **H** GFAP^+^ astrocyte morphology (representative images) and Sholl analysis of branching complexity. *n* = 5 per group. One-Way ANOVA, Two-Way ANOVA, Mean ± SEM. **I** Western Blot analysis of inflammatory cytokine expression in brain tissue. *n* = 4 per group. Two-Way ANOVA, Mean ± SEM. *P < 0.05, **P < 0.01, ***P < 0.001

Wild-type (WT) and UT-B^−/−^ mice were assigned to control and Urea + 1 d treatment groups (Fig. 5A). Throughout the experiment, UT-B^−/−^ mice displayed improved behavioral states and greater resilience to urea challenge. Open-field and grip strength tests showed increased locomotor activity and muscle strength in UT-B^−/−^ mice relative to WT counterparts, although rotarod performance indicated slightly impaired coordination (Fig. 5B and C). Histological analysis further supported a protective phenotype in UT-B-deficient mice. Compared to WT mice, UT-B^−/−^ animals exhibited smaller infarct volumes, attenuated blood-brain barrier disruption, and reduced necrotic and inflammatory cell infiltration (Fig. 5D and F). Notably, although UT-B^−/−^ mice showed a slight increase in brain urea concentration (Fig. 5G), GFAP expression in the ischemic penumbra was lower, and the complexity of astrocytic processes was reduced, indicating suppressed astrocyte activation (Fig. 5H). Moreover, the expression levels of A1-related pro-inflammatory factors and chemokines were not significantly elevated (Fig. 5I; Fig. S4B), demonstrating resistance to urea-driven inflammation.

**Fig. 6:**
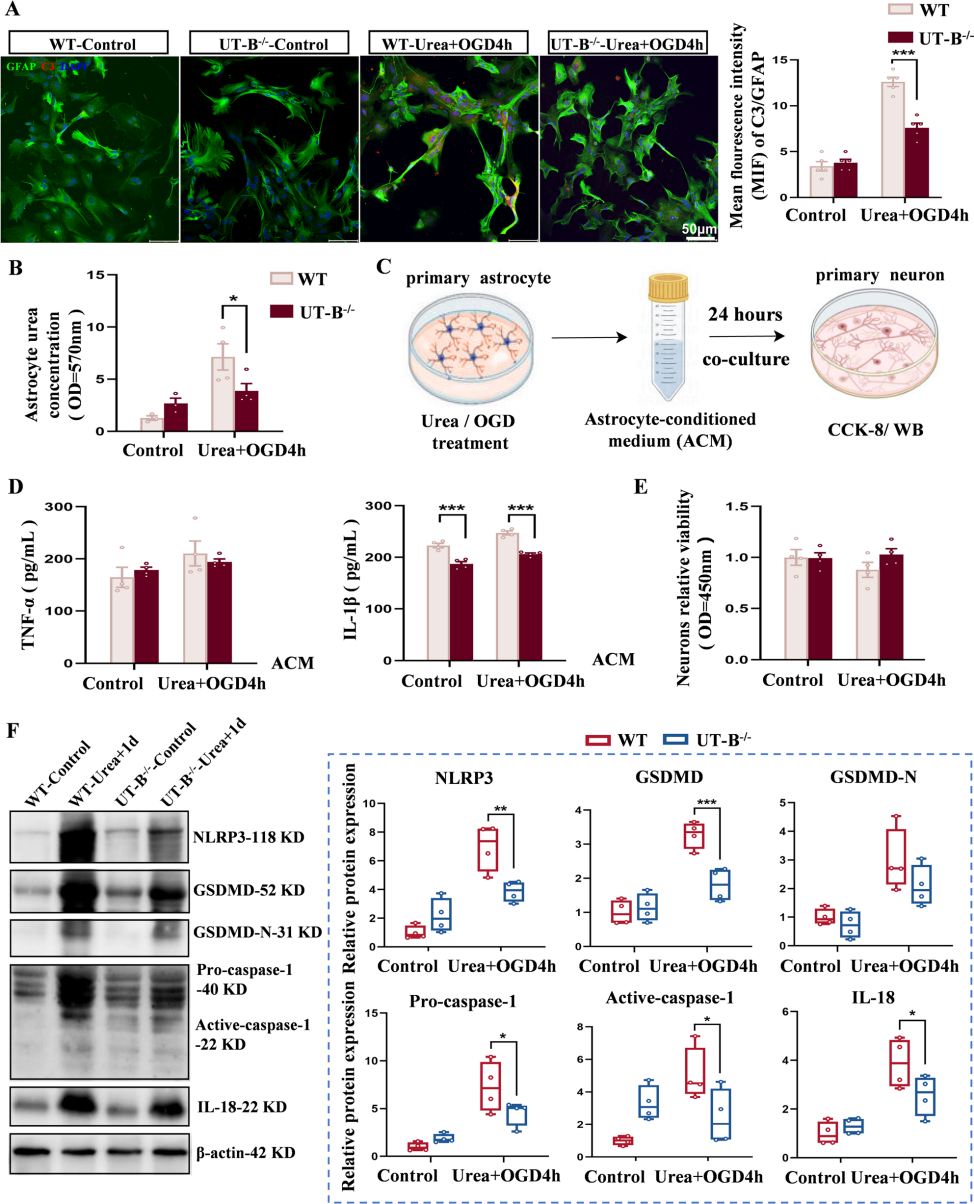
Polarization of neurotoxic astrocytes is suppressed and neuronal activity enhanced in UT-B^−/−^ mice. **A** Immunofluorescence co-staining of C3 (red) and GFAP (green) in astrocytes. Scale bar = 50 μm. *n* = 5 per group. Two-Way ANOVA, Mean ± SEM. **B** Urea concentrations measured in astrocytes from four experimental groups. *n* = 4 per group. Two-Way ANOVA, Mean ± SEM. **C** Astrocyte–neuron co-culture model established using ACM from different groups. **D** ELISA quantification of TNF-α and IL-1β levels in ACM. *n* = 4 per group. Two-Way ANOVA, Mean ± SEM. **E** Neuronal activity recovery following exposure to ACM from UT-B⁻/⁻ astrocytes. *n* = 4 per group. Two-Way ANOVA, Mean ± SEM. **F** Western Blot analysis of pyroptosis-related proteins in neurons across groups. *n* = 4 per group. Two-Way ANOVA, Mean ± SEM. *P < 0.05, **P < 0.01, ***P < 0.001

Consistent findings were obtained in vitro. UT-B protein was nearly undetectable in primary astrocytes from UT-B^−/−^ mice (Fig. S4C). Upon exposure to urea and OGD, these astrocytes exhibited markedly reduced expression of A1-specific markers and chemokines (Fig. 6A; Fig. S4D, S4E), while A2-associated genes showed partial restoration (Fig. S4F). Urea uptake assays confirmed a diminished capacity for UT-B^−/−^ astrocytes to internalize extracellular urea (Fig. 6B), supporting the role of UT-B as a critical transporter mediating urea-induced A1 polarization. ELISA analyses of ACM from these astrocytes showed no increase in TNF-α or IL-1β levels following stimulation (Fig. 6C and D). Consequently, neurons cultured with ACM from UT-B^−/−^ astrocytes demonstrated improved viability (Fig. 6E) and significantly reduced expression of pyroptosis-related proteins (Fig. 6F).

To complement the genetic approach, we employed pharmacological inhibition using UT-B-IN-1, a potent and reversible UT-B inhibitor that binds the intracellular urea-binding site [55]. Dose–response analysis determined 200 nM as the optimal concentration for in vitro inhibition (Fig. S4G-S4I). At this concentration, UT-B-IN-1 significantly reduced C3 protein levels while enhancing expression of A2-related genes (Fig. S5A, S5B). Conditioned medium from Urea + UT-B-IN-1 + OGD-treated astrocytes exhibited reduced inflammatory cytokine content (Fig. S5C), and neurons exposed to this medium maintained higher viability (Fig. S5D). In vivo co-infusion of 160 mM urea with 200 nM UT-B-IN-1 in ischemic mice reproduced the neuroprotective phenotype observed in UT-B^−/−^ mice, with reduced infarct volumes and attenuated inflammatory responses (Fig. S5E-S5K).

Together, these findings demonstrate that both genetic ablation and pharmacological inhibition of UT-B effectively prevent the entry of urea into astrocytes, suppress A1 astrocyte polarization, and protect neurons from inflammatory injury. These results highlight UT-B as a key mediator of urea-induced neurotoxicity and suggest that targeting astrocytic urea transport represents a promising therapeutic strategy for mitigating ischemic brain injury.

### Urea activates the Hippo pathway molecule Wwtr1 and exacerbates astrocytic DNA damage

Our preceding findings identified UT-B as a critical mediator in urea-induced neurotoxicity, acting through astrocytic uptake of urea and subsequent inflammatory responses. However, the intracellular molecular mechanisms underlying urea-induced polarization of reactive astrocytes remain poorly defined. To investigate this, we performed quantitative proteomic analysis on primary astrocytes subjected to urea and/or OGD treatments.

**Fig. 7:**
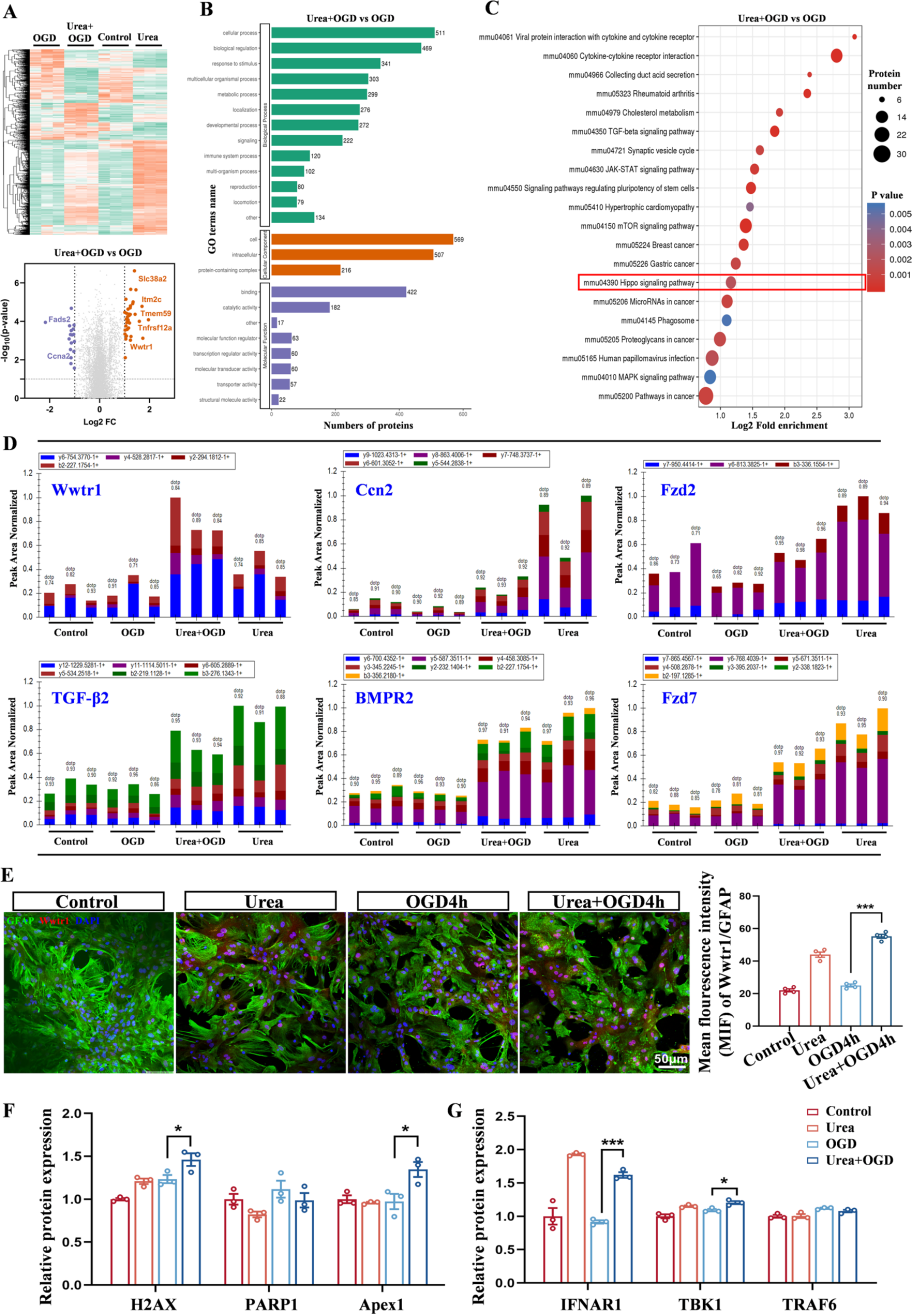
Urea activates the Hippo pathway molecule Wwtr1 and exacerbates astrocytic DNA damage. **A** Proteomic heatmap and volcano plot showing distinct protein expression profiles in astrocytes treated with OGD or Urea + OGD. *n* = 3 per group. **B** Gene Ontology (GO) analysis of differentially expressed proteins between the OGD and Urea + OGD groups. *n* = 3 per group. **C** KEGG pathway enrichment analysis of the same protein set. *n* = 3 per group. **D** Parallel Reaction Monitoring (PRM) quantification of six Hippo pathway-related proteins. *n* = 3 per group. **E** Immunofluorescence co-staining of Wwtr1 (red) and GFAP (green) in astrocytes. Scale bar = 50 μm. *n* = 4 per group. One-Way ANOVA, Mean ± SEM. **F** Proteomic analysis identified DNA damage-related proteins, including H2AX, PARP1, and Apex1. *n* = 3 per group. One-Way ANOVA, Mean ± SEM. **G** Differential protein screening revealed upregulation of cGAS–STING pathway effectors IFNAR1, TBK1, and TRAF6. *n* = 3 per group. One-Way ANOVA, Mean ± SEM. *P < 0.05, ***P < 0.001

Proteomic heatmap clustering revealed distinct protein expression profiles across the four experimental groups. Notably, comparison between the Urea + OGD and OGD-only groups revealed over 600 differentially expressed proteins, indicating that urea substantially alters the proteome of astrocytes under ischemic-mimetic conditions (Fig. 7A). Gene Ontology (GO) analysis classified these proteins predominantly within categories related to “cellular processes” and “biological regulation”, with molecular functions enriched for “binding” and “catalytic activity” (Fig. 7B). Further KEGG pathway enrichment analysis identified several key signaling cascades associated with neuroinflammation, including the mTOR, JAK-STAT, and MAPK pathways [56–59]. Intriguingly, the Hippo signaling pathway-primarily known for its role in regulating cell proliferation, differentiation, and organ size-also emerged as significantly enriched (Fig. 7C), despite having no previously established connection to astrocyte polarization.

To explore this further, we employed parallel reaction monitoring (PRM) to quantify expression of six Hippo pathway-associated proteins. Among them, the transcriptional co-activator Wwtr1 (also known as TAZ) was markedly elevated in the Urea + OGD group compared to OGD group (Fig. 7D). Subsequent Western Blot analysis demonstrated that urea treatment increased Wwtr1 protein expression levels in OGD-intervened astrocytes (Fig. S6A). Immunofluorescence staining further demonstrated nuclear translocation of Wwtr1 (Fig. 7E), indicating its possible functional activation in response to urea exposure. Wwtr1 is a core effector of the Hippo pathway, regulating gene expression by interacting with other nuclear transcription factors, and is known to play roles in proliferation and metabolic control [60]. Previous studies have also implicated aberrant Wwtr1 activation in DNA damage and pro-inflammatory responses in various tissue contexts [33, 61]. Our proteomic screen further revealed altered expression of several DNA damage–associated proteins, including H2AX, PARP1, and Apex1 [62–65] (Fig. 7F). Given that nuclear DNA damage can act as an alarmin, capable of initiating innate immune responses via the cGAS–STING signaling axis [66, 67], we next examined key effectors of this pathway. Notably, IFNAR1, TBK1, and TRAF6-downstream components of cGAS-STING signaling [68–70] -were significantly upregulated in the Urea + OGD group (Fig. 7G), suggesting engagement of DNA damage-driven inflammatory mechanisms. Together, these findings delineate a previously unrecognized molecular cascade linking urea accumulation to reactive astrocyte polarization.

Specifically, our data support a model in which urea activates the Hippo pathway effector Wwtr1, thereby promoting DNA damage and triggering a cGAS-STING-dependent inflammatory response. This cascade may serve as a central mechanism by which urea aggravates neuroinflammation and injury following ischemic stroke.

### Urea induces neurotoxic astrocyte polarization via the Wwtr1-DNA damage pathway

Building upon our proteomic findings, we sought to determine whether the Hippo pathway effector Wwtr1 mediates urea-induced neurotoxic polarization of astrocytes through regulation of the DNA damage response pathway under ischemic stroke pathological conditions. First, immunofluorescence staining revealed that compared with the control group, the expression of Wwtr1 and the DNA double-strand break marker γ-H2AX was significantly increased in the brains of WT-Urea + 1 d mice, and both showed strong co-localization with GFAP. After UT-B gene knockout, the expression of Wwtr1 and γ-H2AX in astrocytes decreased synchronously (Fig. S6B-S6E). Notably, in vitro experiments yielded consistent results: loss of UT-B protein function markedly suppressed Wwtr1 expression and attenuated the associated DNA damage response. Compared to WT controls, UT-B^−/−^ astrocytes exhibited reduced Wwtr1 levels and impaired nuclear localization following Urea + OGD4h treatment (Fig. S6F). Consistently, the expression of γ-H2AX was significantly diminished in UT-B^−/−^ astrocytes (Fig. S6G). Parallel experiments using the UT-B inhibitor reproduced these effects (Fig. S6H, S6I), confirming that UT-B-mediated urea transport is required for Wwtr1 activation and subsequent DNA damage signaling.

**Fig. 8:**
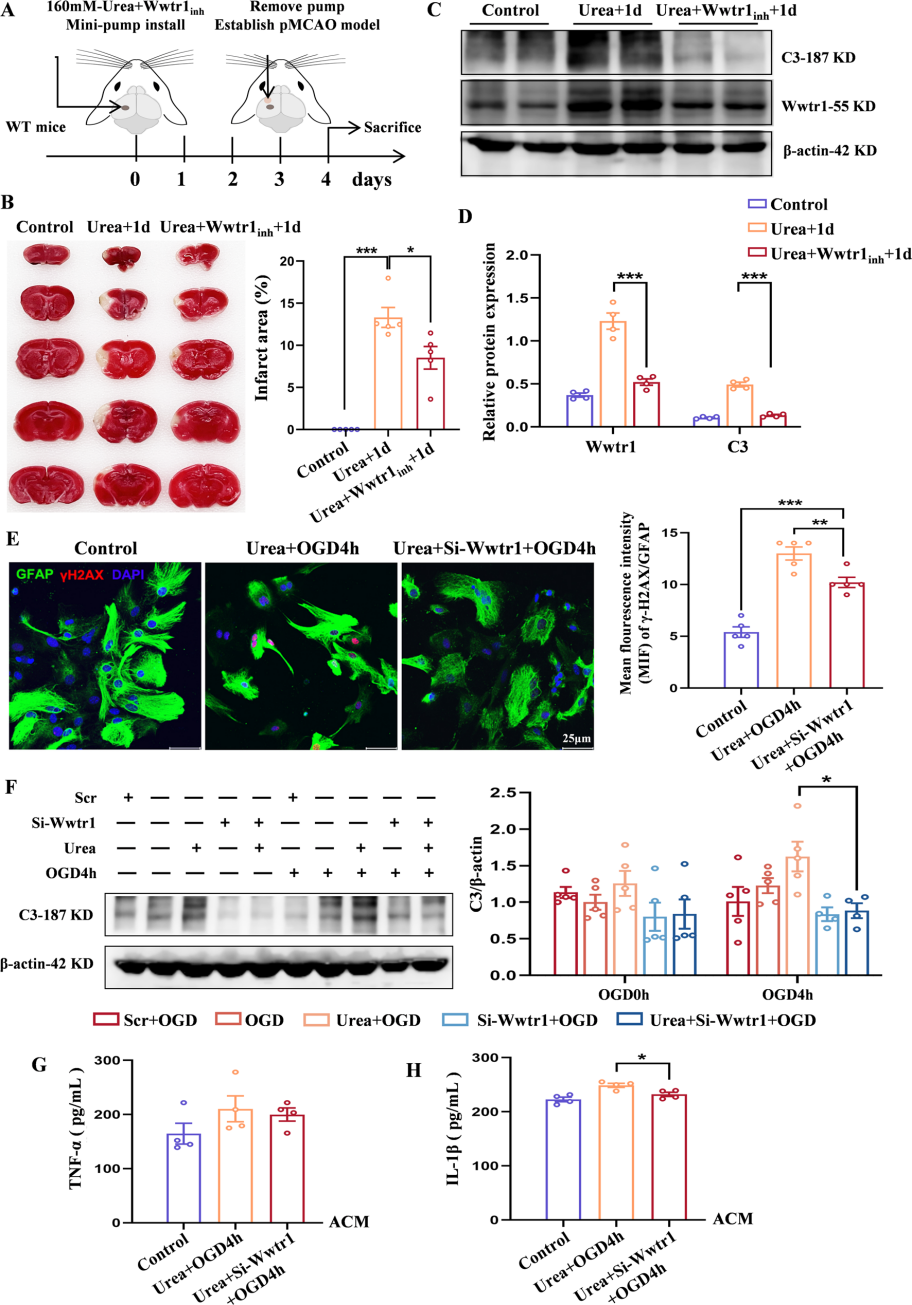
Urea induces neurotoxic astrocyte polarization via the Wwtr1–DNA damage pathway. **A** Experimental setup of the Minipump (160 mM urea + 10 mg/kg EMT inhibitor-1) + pMCAO (1 day) model. **B** TTC staining showing reduced infarct size in Urea + Wwtr1_inh_ + 1 d group mice brains. *n* = 5 per group. One-Way ANOVA, Mean ± SEM. **C**, **D** Western Blot analysis of Wwtr1 and C3 proteins in brains across groups. *n* = 4 per group. One-Way ANOVA, Mean ± SEM. **E** Immunofluorescence co-staining of γ-H2AX (red) and GFAP (green) assessing DNA damage following Wwtr1 silencing. Scale bar = 25 μm. *n* = 5 per group. One-Way ANOVA, Mean ± SEM. **F** Western Blot showing reduced C3 expression after Wwtr1 knockdown. *n* = 5 per group. One-Way ANOVA, Mean ± SEM. **G**, **H** ELISA quantification of TNF-α and IL-1β levels in ACM. *n* = 4 per group. One-Way ANOVA, Mean ± SEM. *P < 0.05, **P < 0.01, ***P < 0.001

To determine whether Wwtr1 promotes A1 astrocyte polarization by mediating DNA damage, we first treated mice with EMT inhibitor-1 (which induces Wwtr1 degradation via activation of Hippo kinases Mst/Lats and AMPK) in combination with urea. Osmotic pump delivery (Fig. 8A) revealed that the Urea + Wwtr1_inh_ + 1 d group exhibited significantly reduced cerebral infarct volume compared to Urea + 1 d group (Fig. 8B), with decreased C3 protein expression in brain tissue following Wwtr1 downregulation (Fig. 8C and D). qPCR analysis further confirmed marked downregulation of A1 markers (*C3*,* IL-1β*,* IFN-γ*) and pro-inflammatory chemokines (*CCL2*,* CXCL10*) (Fig. S7A, S7B), while A2 neuroprotective genes (*Emp1*,* CD109*,* IL-10*) were upregulated (Fig. S7C). Notably, DNA damage-related genes (*IFNAR1*,* TRAF6*) also showed Wwtr1-dependent suppression (Fig. S7D).

To validate these findings, we silenced its expression in primary astrocytes using siRNA. Western Blot analysis verified effective knockdown of Wwtr1 in the si-Wwtr1 group compared to scrambled controls (Fig. S7E). Concomitantly, expression of γ-H2AX was significantly reduced (Fig. 8E), indicating a suppression of urea-induced DNA damage. Importantly, UT-B expression remained unchanged in si-Wwtr1 cells (Fig. S7F), confirming that the observed effects were specific to Wwtr1 knockdown. Further analysis revealed that Wwtr1 silencing attenuated astrocyte polarization toward the neurotoxic A1 phenotype. Western Blot showed a marked reduction in C3 protein levels in astrocytes from the Urea + si-Wwtr1 + OGD group (Fig. 8F), while ELISA analysis of conditioned medium indicated decreased secretion of pro-inflammatory cytokines TNF-α and IL-1β (Fig. 8G and H).

This part demonstrates that urea activates Wwtr1 via UT-B-dependent transport, leading to enhanced DNA damage and promoting polarization of astrocytes into the neurotoxic A1 subtype. Wwtr1 thus serves as a central mediator linking urea accumulation to astrocyte-driven neuroinflammation and neuronal injury following ischemic stroke.

## Discussion

Inflammatory responses are inherent throughout an organism’s lifespan, and the CNS is no exception. Although the BBB has long been considered a structural and immunological shield that maintains brain immune privilege [71, 72], neuroinflammation can persist in the absence of pathogens [73, 74]. Recent studies have identified several endogenous metabolites that modulate neuroinflammatory processes, either directly or indirectly by influencing microglial and astrocytic activation, cytokine release, and oxidative stress [75–77]. In this study, we first revealed that urea—a well-established end-product of nitrogen metabolism-as a previously unrecognized endogenous risk signal contributing to ischemic stroke pathology. By employing a combination of in vivo and in vitro models, we show that ischemic injury leads to a rapid accumulation of urea in the affected brain hemisphere. Mechanistically, this accumulation is followed by astrocytic uptake of urea through the urea transporter UT-B, which in turn activates the Hippo pathway effector Wwtr1, triggers nuclear DNA damage, induces A1-type neurotoxic astrocyte polarization, and ultimately leads to neuronal pyroptosis. Importantly, the elevation of urea levels was tightly correlated with the severity of ischemic injury, suggesting its relevance as both a biomarker and a pathogenic mediator in stroke.

Previous studies have shown that urea levels are elevated in the brains of patients with Alzheimer’s and Huntington’s diseases, suggesting a potential link between excessive urea accumulation and CNS pathology. In both clinical specimens and pMCAO mouse models, we observed significantly elevated urea levels in ischemic brain tissue, particularly within the ischemic core, accompanied by upregulation of urea cycle enzymes. These findings suggest local metabolic activation of the urea cycle, possibly as a response to energy imbalance and nitrogen accumulation in the ischemic tissue. Notably, the infarct volume positively correlated with urea levels. This data suggest that elevated urea is not merely a metabolic by-product, but a pathogenic factor contributing to ischemic brain damage. In support of this concept, the spatial distribution of urea within the ischemic hemisphere was notably heterogeneous, with significantly higher concentrations detected in the ischemic core compared to the penumbra. This regional pattern of accumulation closely paralleled the zone of maximal tissue necrosis, suggesting that urea may originate from metabolically compromised or dying neural cells. Moreover, the observed upregulation of urea cycle enzymes, including ASS1, and ASL, in ischemic brain tissue implies a compensatory metabolic shift toward urea synthesis under stress conditions. These changes not only reflect local nitrogen dysregulation but also support the interpretation that ischemia-induced urea production may actively contribute to pathological signaling rather than merely reflecting tissue breakdown.

The activation of A1-type astrocytes plays a pivotal role in sterile inflammation of the CNS. In our models, exogenous urea delivery or ischemic injury alone induced moderate astrocyte activation, but their combination resulted in a pronounced shift toward the A1 phenotype, with increased expression of C3 and pro-inflammatory cytokines (TNF-α, IL-1β), and suppression of A2 markers. This polarization was accompanied by increased expression of CCL2 and CXCL10, chemokines that facilitate peripheral immune cell infiltration. Our astrocyte–neuron co-culture system further confirmed the indirect neurotoxic effects of urea, as neurons exposed to astrocyte-conditioned medium from the Urea + OGD group exhibited reduced viability and activation of pyroptotic markers (NLRP3, GSDMD, Caspase-1), despite negligible changes in apoptosis markers Bax and Bcl-2. These observations suggest that neuronal death in this context is driven predominantly by urea-induced astrocyte inflammation, not by direct toxicity of urea itself.

This hypothesis was supported by comparative expression analysis of UT-B in neurons and astrocytes. Astrocytes expressed significantly higher levels of UT-B, while neurons displayed minimal expression [78] (Fig. S7G), and thus likely lacked the capacity for effective urea uptake. This provides a mechanistic explanation for the cell type-specific effects of urea, emphasizing astrocytes as the primary responders and amplifiers of urea-mediated neurotoxicity. This cell type specificity is further reflected in functional outcomes. While high levels of urea in astrocyte-conditioned medium (ACM) led to the emergence of a strongly pro-inflammatory astrocyte phenotype, the direct application of urea alone did not significantly affect neuronal viability. In contrast, neurons exposed to ACM derived from urea- and OGD-treated astrocytes exhibited reduced survival and upregulated pyroptosis-related proteins. This reinforces the idea that urea’s deleterious effects are not exerted through direct neuronal toxicity, but rather via astrocyte-mediated signaling cascades that shape the neuroinflammatory environment.

Our data further UT-B as a critical regulatory node in this process. Ischemic stress markedly increased UT-B expression in astrocytes, and both genetic knockout and pharmacological inhibition of UT-B (via UT-B-IN-1) attenuated A1 astrocyte polarization, reduced inflammatory cytokine production, and mitigated neuronal injury. Paradoxically, UT-B^−/−^ mice exhibited higher cerebral urea levels than wild-type controls, but less neurological damage. This discrepancy underscores the importance of intracellular compartmentalization of urea, rather than its absolute extracellular concentration, in mediating its harmful effects. UT-B-dependent transport into astrocytes appears to be a prerequisite for urea to exert its pro-inflammatory functions-highlighting UT-B as a tractable therapeutic target. In behavioral terms, the functional benefits of UT-B inhibition were evident in both UT-B^−/−^ mice and in animals treated with the pharmacological inhibitor UT-B-IN-1. These mice showed significantly improved motor activity and reduced neurological deficits following ischemic insult, compared to their wild-type or untreated counterparts. These phenotypic improvements closely mirrored molecular signatures, including suppressed expression of C3, TNF-α, and IL-1β in astrocytes, and reduced pyroptosis in co-cultured neurons. Together, these results demonstrate that targeting astrocytic urea transport can confer both molecular and functional protection against ischemic injury.

Beyond its transport role, urea initiates downstream signaling pathways that amplify neuroinflammation. Proteomic and PRM analyses revealed significant enrichment of Hippo pathway components in urea-treated astrocytes, with particular upregulation of Wwtr1, a key transcriptional coactivator. We demonstrate that Wwtr1 is translocated to the nucleus following urea uptake and contributes to DNA damage, as evidenced by increased γ-H2AX expression. Wwtr1 knockdown via siRNA and pharmacological inhibition (EMT inhibitor-1) not only reduced DNA damage but also reversed the A1 polarization profile and cytokine production, confirming the central role of the UT-B-Wwtr1-DNA damage axis in astrocyte reprogramming. This work, extends the functional scope of the Hippo pathway from classical regulation of organ size to modulation of neuroinflammation in response to metabolic stress. Thus, our study not only reveals a novel upstream pathway regulating A1 astrocyte activation but also integrates metabolic, inflammatory, and transcriptional pathways into a unified model of urea-induced CNS injury. In summary, we provide comprehensive evidence that urea acts as a risk molecule in ischemic stroke. Upon entering astrocytes through UT-B, urea activates the Hippo-Wwtr1 pathway, induces DNA damage, promotes neurotoxic astrocyte polarization, and indirectly drives neuronal pyroptosis. This urea-UT-B-Wwtr1 axis offers a new paradigm for understanding the role of endogenous metabolites in CNS injury and inflammation.

However, this study also has several limitations. First, the precise origin of urea in ischemic brain tissue has not been fully elucidated. Future studies could employ stable isotope labeling techniques (e.g., ^13 C or ^15 N) to track urea dynamics in vivo, which would provide more direct evidence for elucidating the pathophysiological role of urea. Moreover, although UT-B inhibition has demonstrated promising neuroprotective effects in mouse models, its clinical application still faces significant challenges. Given that UT-B is widely expressed in peripheral organs such as the kidneys and plays a crucial role in urine concentration and other key physiological processes, systemic blockade of UT-B may lead to adverse effects such as diabetes insipidus, electrolyte imbalances, and disruption of systemic nitrogen metabolism [79, 80]. Hence, the development of CNS-targeted UT-B-specific inhibitors or delivery systems will be critical for clinical translation. Finally, dysregulated urea metabolism is closely associated with various chronic neurological disorders. Future research could validate the generalizability of our findings in other neurological disease models, which would help expand the theoretical significance and potential therapeutic value of the urea metabolic pathway in neurological diseases.

## Supplementary Information

Supplementary Material 1.

## Data Availability

The data in our study are available from the corresponding author upon reasonable request.

## Abbreviations

BBB: Blood-brain barrier
CCK-8: Cell Counting Kit-8
CNS: Central nervous system
DAPI: 4′,6-diamidino-2-phenylindole dihydrochloride
EB: Evans blue
γ-H2AX: Gamma-Histone H2A family member X
OGD: Oxygen-Glucose deprivation
pMCAO: Permanent middle cerebral artery occlusion
TTC: 2，3，5-Triphenyltetrazolium chloride
UT: Urea transporter
Wwtr1: WW domain containing transcription regulator 1

## References

[1] Huang H, Oo TT, Apaijai N, Chattipakorn N, Chattipakorn SC. An updated review of mitochondrial transplantation as a potential therapeutic strategy against cerebral ischemia and cerebral ischemia/Reperfusion injury. Mol Neurobiol. 2023;60(4):1865–83. 36595193 10.1007/s12035-022-03200-y

[2] Hankey GJ. Secondary stroke prevention. Lancet Neurol. 2014;13(2):178–94. 24361114 10.1016/S1474-4422(13)70255-2

[3] Hong X, Jian Y, Ding S, Zhou J, Zheng X, Zhang H, Zhou B, Zhuang C, Wan J, Tong X. Kir4.1 channel activation in NG2 glia contributes to remyelination in ischemic stroke. EBioMedicine. 2023;87:104406. 36527899 10.1016/j.ebiom.2022.104406 PMC9791134

[4] Saini V, Guada L, Yavagal DR. Global epidemiology of stroke and access to acute ischemic stroke interventions. Neurology. 2021;97(20 Suppl 2):S6–16. 34785599 10.1212/WNL.0000000000012781

[5] Guan X, Zhu S, Song J, Liu K, Liu M, Xie L, Wang Y, Wu J, Xu X, Pang T. Microglial CMPK2 promotes neuroinflammation and brain injury after ischemic stroke. Cell Rep Med. 2024;5(5):101522. 38701781 10.1016/j.xcrm.2024.101522 PMC11148565

[6] Candelario-Jalil E, Dijkhuizen RM, Magnus T. Neuroinflammation, Stroke, Blood-Brain barrier Dysfunction, and imaging modalities. Stroke. 2022;53(5):1473–86. 35387495 10.1161/STROKEAHA.122.036946 PMC9038693

[7] Banjara M, Ghosh C. Sterile neuroinflammation and strategies for therapeutic intervention. Int J Inflam. 2017;2017:8385961. 10.1155/2017/8385961 PMC5239986 28127491

[8] Matsuda S, Umeda M, Kato H, Araki T. Glial damage after transient focal cerebral ischemia in rats. J Mol Neurosci. 2009;38(2):220–6. 19051061 10.1007/s12031-008-9165-4

[9] Chen J, Jin K, Chen M, Pei W, Kawaguchi K, Greenberg DA, Simon RP. Early detection of DNA strand breaks in the brain after transient focal ischemia: implications for the role of DNA damage in apoptosis and neuronal cell death. J Neurochem. 1997;69(1):232–45. 9202315 10.1046/j.1471-4159.1997.69010232.x

[10] Shambaugh GE 3. Urea biosynthesis I. The Urea cycle and relationships to the citric acid cycle. Am J Clin Nutr. 1977;30(12):2083–7. 10.1093/ajcn/30.12.2083 337792

[11] Wang H, Ran J, Jiang T, Urea. Subcell Biochem. 2014;73:7–29. 25298336 10.1007/978-94-017-9343-8_2

[12] Pundir CS, Jakhar S, Narwal V. Determination of Urea with special emphasis on biosensors: A review. Biosens Bioelectron. 2019;123:36–50. 30308420 10.1016/j.bios.2018.09.067

[13] Morris SM Jr. Regulation of enzymes of the Urea cycle and arginine metabolism. Annu Rev Nutr. 2002;22:87–105. 12055339 10.1146/annurev.nutr.22.110801.140547

[14] Ju YH, Bhalla M, Hyeon SJ, Oh JE, Yoo S, Chae U, Kwon J, Koh W, Lim J, Park YM, et al. Astrocytic Urea cycle detoxifies Aβ-derived ammonia while impairing memory in alzheimer’s disease. Cell Metab. 2022;34(8):1104–e11201108. 35738259 10.1016/j.cmet.2022.05.011

[15] Handley RR, Reid SJ, Brauning R, Maclean P, Mears ER, Fourie I, Patassini S, Cooper GJS, Rudiger SR, McLaughlan CJ, et al. Brain Urea increase is an early huntington’s disease pathogenic event observed in a prodromal Transgenic sheep model and HD cases. Proc Natl Acad Sci U S A. 2017;114(52):E11293–302. 29229845 10.1073/pnas.1711243115 PMC5748180

[16] Ecanow B, Gold BH, Tunkunas P. Serum albumin and Urea during States of anxiety and depression. JAMA. 1973;226(3):356. 4740944

[17] Wang H, Huang B, Wang W, Li J, Chen Y, Flynn T, Zhao M, Zhou Z, Lin X, Zhang Y, et al. High Urea induces depression and LTP impairment through mTOR signalling suppression caused by carbamylation. EBioMedicine. 2019;48:478–90. 31628020 10.1016/j.ebiom.2019.09.049 PMC6838447

[18] Smith CP. Mammalian Urea transporters. Exp Physiol. 2009;94(2):180–5. 19028811 10.1113/expphysiol.2008.043042

[19] Shayakul C, Clémençon B, Hediger MA. The Urea transporter family (SLC14): physiological, pathological and structural aspects. Mol Aspects Med. 2013;34(2–3):313–22. 23506873 10.1016/j.mam.2012.12.003

[20] Smith CP, Fenton RA. Genomic organization of the mammalian SLC14a2 Urea transporter genes. J Membr Biol. 2006;212(2):109–17. 17264986 10.1007/s00232-006-0870-z

[21] Yu L, Liu T, Fu S, Li L, Meng X, Su X, Xie Z, Ren J, Meng Y, Lv X, et al. Physiological functions of Urea transporter B. Pflugers Arch. 2019;471(11–12):1359–68. 31734718 10.1007/s00424-019-02323-x PMC6882768

[22] Li X, Ran J, Zhou H, Lei T, Zhou L, Han J, Yang B. Mice lacking Urea transporter UT-B display depression-like behavior. J Mol Neurosci. 2012;46(2):362–72. 21750947 10.1007/s12031-011-9594-3

[23] Berger UV, Tsukaguchi H, Hediger MA. Distribution of mRNA for the facilitated Urea transporter UT3 in the rat nervous system. Anat Embryol (Berl). 1998;197(5):405–14. 9623675 10.1007/s004290050152

[24] Ma S, Meng Z, Chen R, Guan KL. The Hippo pathway: biology and pathophysiology. Annu Rev Biochem. 2019;88:577–604. 30566373 10.1146/annurev-biochem-013118-111829

[25] Fu M, Hu Y, Lan T, Guan KL, Luo T, Luo M. The Hippo signalling pathway and its implications in human health and diseases. Signal Transduct Target Ther. 2022;7(1):376. 36347846 10.1038/s41392-022-01191-9 PMC9643504

[26] Zhong Z, Jiao Z, Yu FX. The Hippo signaling pathway in development and regeneration. Cell Rep. 2024;43(3):113926. 38457338 10.1016/j.celrep.2024.113926

[27] Moya IM, Castaldo SA, Van den Mooter L, Soheily S, Sansores-Garcia L, Jacobs J, Mannaerts I, Xie J, Verboven E, Hillen H, et al. Peritumoral activation of the Hippo pathway effectors YAP and TAZ suppresses liver cancer in mice. Science. 2019;366(6468):1029–34. 31754005 10.1126/science.aaw9886

[28] Maugeri-Saccà M, De Maria R. The Hippo pathway in normal development and cancer. Pharmacol Ther. 2018;186:60–72. 29305295 10.1016/j.pharmthera.2017.12.011

[29] Totaro A, Panciera T, Piccolo S. YAP/TAZ upstream signals and downstream responses. Nat Cell Biol. 2018;20(8):888–99. 30050119 10.1038/s41556-018-0142-z PMC6186418

[30] Wang KC, Yeh YT, Nguyen P, Limqueco E, Lopez J, Thorossian S, Guan KL, Li YJ, Chien S. Flow-dependent YAP/TAZ activities regulate endothelial phenotypes and atherosclerosis. Proc Natl Acad Sci U S A. 2016;113(41):11525–30. 27671657 10.1073/pnas.1613121113 PMC5068257

[31] Mia MM, Cibi DM, Abdul Ghani SAB, Song W, Tee N, Ghosh S, Mao J, Olson EN, Singh MK. YAP/TAZ deficiency reprograms macrophage phenotype and improves infarct healing and cardiac function after myocardial infarction. PLoS Biol. 2020;18(12):e3000941. 33264286 10.1371/journal.pbio.3000941 PMC7735680

[32] Wang X, Zeldin S, Shi H, Zhu C, Saito Y, Corey KE, Osganian SA, Remotti HE, Verna EC, Pajvani UB, et al. TAZ-induced Cybb contributes to liver tumor formation in non-alcoholic steatohepatitis. J Hepatol. 2022;76(4):910–20. 34902531 10.1016/j.jhep.2021.11.031 PMC8934258

[33] Wang X, Zheng Z, Caviglia JM, Corey KE, Herfel TM, Cai B, Masia R, Chung RT, Lefkowitch JH, Schwabe RF, et al. Hepatocyte TAZ/WWTR1 promotes inflammation and fibrosis in nonalcoholic steatohepatitis. Cell Metab. 2016;24(6):848–62. 28068223 10.1016/j.cmet.2016.09.016 PMC5226184

[34] Kunze R, Fischer S, Marti HH, Preissner KT. Brain alarm by self-extracellular nucleic acids: from neuroinflammation to neurodegeneration. J Biomed Sci. 2023;30(1):64. 37550658 10.1186/s12929-023-00954-y PMC10405513

[35] Gulen MF, Samson N, Keller A, Schwabenland M, Liu C, Glück S, Thacker VV, Favre L, Mangeat B, Kroese LJ, et al. cGAS-STING drives ageing-related inflammation and neurodegeneration. Nature. 2023;620(7973):374–80. 37532932 10.1038/s41586-023-06373-1 PMC10412454

[36] Zhang W, Sun HS, Wang X, Dumont AS, Liu Q. Cellular senescence, DNA damage, and neuroinflammation in the aging brain. Trends Neurosci. 2024;47(6):461–74. 38729785 10.1016/j.tins.2024.04.003

[37] Giordano AMS, Luciani M, Gatto F, Abou Alezz M, Beghè C, Della Volpe L, Migliara A, Valsoni S, Genua M, Dzieciatkowska M, et al. DNA damage contributes to neurotoxic inflammation in Aicardi-Goutières syndrome astrocytes. J Exp Med. 2022;219(4):e20211121. 10.1084/jem.20211121 PMC8916121 35262626

[38] Yang B, Bankir L, Gillespie A, Epstein CJ, Verkman AS. Urea-selective concentrating defect in Transgenic mice lacking Urea transporter UT-B. J Biol Chem. 2002;277(12):10633–7. 11792714 10.1074/jbc.M200207200

[39] Klein JD, Sands JM, Qian L, Wang X, Yang B. Upregulation of Urea transporter UT-A2 and water channels AQP2 and AQP3 in mice lacking Urea transporter UT-B. J Am Soc Nephrol. 2004;15(5):1161–7. 15100356 10.1097/01.asn.0000125617.19799.72

[40] Bandera E, Botteri M, Minelli C, Sutton A, Abrams KR, Latronico N. Cerebral blood flow threshold of ischemic penumbra and infarct core in acute ischemic stroke: a systematic review. Stroke. 2006;37(5):1334–9. 16574919 10.1161/01.STR.0000217418.29609.22

[41] Ermine CM, Bivard A, Parsons MW, Baron JC. The ischemic penumbra: from concept to reality. Int J Stroke. 2021;16(5):497–509. 33818215 10.1177/1747493020975229

[42] Opie EL. Changes in the osmotic activity of liver and of kidney tissue caused by passage of sodium chloride, urea, and some other substances into cells. J Exp Med. 1956;103(3):351–62. 13295491 10.1084/jem.103.3.351 PMC2136586

[43] Liu A, Yu L, Li X, Zhang K, Zhang W, So KF, Tissir F, Qu Y, Zhou L. Celsr2-mediated morphological polarization and functional phenotype of reactive astrocytes in neural repair. Glia. 2023;71(8):1985–2004. 37186402 10.1002/glia.24378

[44] Zhao N, Xu X, Jiang Y, Gao J, Wang F, Xu X, Wen Z, Xie Y, Li J, Li R, et al. Lipocalin-2 May produce damaging effect after cerebral ischemia by inducing astrocytes classical activation. J Neuroinflammation. 2019;16(1):168. 31426811 10.1186/s12974-019-1556-7 PMC6699078

[45] Giovannoni F, Quintana FJ. The role of astrocytes in CNS inflammation. Trends Immunol. 2020;41(9):805–19. 32800705 10.1016/j.it.2020.07.007 PMC8284746

[46] Wang C, Li L. The critical role of KLF4 in regulating the activation of A1/A2 reactive astrocytes following ischemic stroke. J Neuroinflammation. 2023;20(1):44. 36823628 10.1186/s12974-023-02742-9 PMC9948409

[47] Guo Q, Gobbo D, Zhao N, Zhang H, Awuku NO, Liu Q, Fang LP, Gampfer TM, Meyer MR, Zhao R, et al. Adenosine triggers early astrocyte reactivity that provokes microglial responses and drives the pathogenesis of sepsis-associated encephalopathy in mice. Nat Commun. 2024;15(1):6340. 39068155 10.1038/s41467-024-50466-y PMC11283516

[48] Orchanian SB, Still K, Harris TH, Lodoen MB. Deficiency in astrocyte CCL2 production reduces neuroimmune control of Toxoplasma gondii infection. PLoS Pathog. 2024;20(1):e1011710. 38206985 10.1371/journal.ppat.1011710 PMC10807779

[49] Taub DD, Longo DL, Murphy WJ. Human interferon-inducible protein-10 induces mononuclear cell infiltration in mice and promotes the migration of human T lymphocytes into the peripheral tissues and human peripheral blood lymphocytes-SCID mice. Blood. 1996;87(4):1423–31. 8608232

[50] Novakovic MM, Korshunov KS, Grant RA, Martin ME, Valencia HA, Budinger GRS, Radulovic J, Prakriya M. Astrocyte reactivity and inflammation-induced depression-like behaviors are regulated by Orai1 calcium channels. Nat Commun. 2023;14(1):5500. 37679321 10.1038/s41467-023-40968-6 PMC10485021

[51] Ryu KY, Lee HJ, Woo H, Kang RJ, Han KM, Park H, Lee SM, Lee JY, Jeong YJ, Nam HW, et al. Dasatinib regulates LPS-induced microglial and astrocytic neuroinflammatory responses by inhibiting AKT/STAT3 signaling. J Neuroinflammation. 2019;16(1):190. 31655606 10.1186/s12974-019-1561-x PMC6815018

[52] Patabendige A, Singh A, Jenkins S, Sen J, Chen R. Astrocyte activation in neurovascular damage and repair following ischaemic stroke. Int J Mol Sci. 2021;22(8):4280. 10.3390/ijms22084280 PMC8074612 33924191

[53] Ding ZB, Song LJ, Wang Q, Kumar G, Yan YQ, Ma CG. Astrocytes: a double-edged sword in neurodegenerative diseases. Neural Regen Res. 2021;16(9):1702–10. 33510058 10.4103/1673-5374.306064 PMC8328766

[54] Liu M, Xu Z, Wang L, Zhang L, Liu Y, Cao J, Fu Q, Liu Y, Li H, Lou J, et al. Cottonseed oil alleviates ischemic stroke injury by inhibiting the inflammatory activation of microglia and astrocyte. J Neuroinflammation. 2020;17(1):270. 32917229 10.1186/s12974-020-01946-7 PMC7488511

[55] Yao C, Anderson MO, Zhang J, Yang B, Phuan PW, Verkman AS. Triazolothienopyrimidine inhibitors of Urea transporter UT-B reduce urine concentration. J Am Soc Nephrol. 2012;23(7):1210–20. 22491419 10.1681/ASN.2011070751 PMC3380644

[56] Xian M, Cai J, Zheng K, Liu Q, Liu Y, Lin H, Liang S, Wang S. Aloe-emodin prevents nerve injury and neuroinflammation caused by ischemic stroke via the PI3K/AKT/mTOR and NF-κB pathway. Food Funct. 2021;12(17):8056–67. 34286782 10.1039/d1fo01144h

[57] Angerfors A, Brännmark C, Lagging C, Tai K, Månsby Svedberg R, Andersson B, Jern C, Stanne TM. Proteomic profiling identifies novel inflammation-related plasma proteins associated with ischemic stroke outcome. J Neuroinflammation. 2023;20(1):224. 37794467 10.1186/s12974-023-02912-9 PMC10548608

[58] Jayaraj RL, Azimullah S, Beiram R, Jalal FY, Rosenberg GA. Neuroinflammation: friend and foe for ischemic stroke. J Neuroinflammation. 2019;16(1):142. 31291966 10.1186/s12974-019-1516-2 PMC6617684

[59] Larochelle J, Tishko RJ, Yang C, Ge Y, Phan LT, Gunraj RE, Stansbury SM, Liu L, Mohamadzadeh M, Khoshbouei H, et al. Receptor-interacting protein kinase 2 (RIPK2) profoundly contributes to post-stroke neuroinflammation and behavioral deficits with microglia as unique perpetrators. J Neuroinflammation. 2023;20(1):221. 37777791 10.1186/s12974-023-02907-6 PMC10543871

[60] Koo JH, Guan KL. Interplay between YAP/TAZ and metabolism. Cell Metab. 2018;28(2):196–206. 30089241 10.1016/j.cmet.2018.07.010

[61] Wang X, Cai B, Yang X, Sonubi OO, Zheng Z, Ramakrishnan R, Shi H, Valenti L, Pajvani UB, Sandhu J, et al. Cholesterol stabilizes TAZ in hepatocytes to promote experimental Non-alcoholic steatohepatitis. Cell Metab. 2020;31(5):969–e986967. 32259482 10.1016/j.cmet.2020.03.010 PMC7313619

[62] Mah LJ, El-Osta A, Karagiannis TC. gammaH2AX: a sensitive molecular marker of DNA damage and repair. Leukemia. 2010;24(4):679–86. 20130602 10.1038/leu.2010.6

[63] Ivashkevich A, Redon CE, Nakamura AJ, Martin RF, Martin OA. Use of the γ-H2AX assay to monitor DNA damage and repair in translational cancer research. Cancer Lett. 2012;327(1–2):123–33. 22198208 10.1016/j.canlet.2011.12.025 PMC3329565

[64] Ray Chaudhuri A, Nussenzweig A. The multifaceted roles of PARP1 in DNA repair and chromatin remodelling. Nat Rev Mol Cell Biol. 2017;18(10):610–21. 28676700 10.1038/nrm.2017.53 PMC6591728

[65] Chen Q, Chen T, Xiao H, Wang F, Li C, Hu N, Bao L, Tong X, Feng Y, Xu Y, et al. APEX1 in intestinal epithelium triggers neutrophil infiltration and intestinal barrier damage in ulcerative colitis. Free Radic Biol Med. 2024;225:359–73. 39389211 10.1016/j.freeradbiomed.2024.10.260

[66] Hopfner KP, Hornung V. Molecular mechanisms and cellular functions of cGAS-STING signalling. Nat Rev Mol Cell Biol. 2020;21(9):501–21. 32424334 10.1038/s41580-020-0244-x

[67] Dvorkin S, Cambier S, Volkman HE, Stetson DB. New frontiers in the cGAS-STING intracellular DNA-sensing pathway. Immunity. 2024;57(4):718–30. 38599167 10.1016/j.immuni.2024.02.019 PMC11013568

[68] Liu Y, Wang A, Chen C, Zhang Q, Shen Q, Zhang D, Xiao X, Chen S, Lian L, Le Z, et al. Microglial cGAS-STING signaling underlies glaucoma pathogenesis. Proc Natl Acad Sci U S A. 2024;121(36):e2409493121. 39190350 10.1073/pnas.2409493121 PMC11388346

[69] Luksch H, Stinson WA, Platt DJ, Qian W, Kalugotla G, Miner CA, Bennion BG, Gerbaulet A, Rösen-Wolff A, Miner JJ. STING-associated lung disease in mice relies on T cells but not type I interferon. J Allergy Clin Immunol. 2019;144(1):254–e266258. 30772497 10.1016/j.jaci.2019.01.044 PMC6612314

[70] Oduro PK, Zheng X, Wei J, Yang Y, Wang Y, Zhang H, Liu E, Gao X, Du M, Wang Q. The cGAS-STING signaling in cardiovascular and metabolic diseases: future novel target option for pharmacotherapy. Acta Pharm Sin B. 2022;12(1):50–75. 35127372 10.1016/j.apsb.2021.05.011 PMC8799861

[71] Banks WA. The blood-brain barrier in neuroimmunology: Tales of separation and assimilation. Brain Behav Immun. 2015;44:1–8. 25172555 10.1016/j.bbi.2014.08.007 PMC4275374

[72] Erickson MA, Banks WA. Neuroimmune axes of the Blood-Brain barriers and Blood-Brain interfaces: bases for physiological Regulation, disease States, and Pharmacological interventions. Pharmacol Rev. 2018;70(2):278–314. 29496890 10.1124/pr.117.014647 PMC5833009

[73] Javidi E, Magnus T. Autoimmunity after ischemic stroke and brain injury. Front Immunol. 2019;10:686. 31001280 10.3389/fimmu.2019.00686 PMC6454865

[74] Nakamura K, Sakai S, Tsuyama J, Nakamura A, Otani K, Kurabayashi K, Yogiashi Y, Masai H, Shichita T. Extracellular DJ-1 induces sterile inflammation in the ischemic brain. PLoS Biol. 2021;19(5):e3000939. 34014921 10.1371/journal.pbio.3000939 PMC8136727

[75] Wei L, Yang X, Wang J, Wang Z, Wang Q, Ding Y, Yu A. H3K18 lactylation of senescent microglia potentiates brain aging and alzheimer’s disease through the NFκB signaling pathway. J Neuroinflammation. 2023;20(1):208. 37697347 10.1186/s12974-023-02879-7 PMC10494370

[76] Chang NP, DaPrano EM, Evans WR, Nissenbaum M, McCourt M, Alzate D, Lindman M, Chou TW, Atkins C, Kusnecov AW, et al. Neuronal DAMPs exacerbate neurodegeneration via astrocytic RIPK3 signaling. bioRxiv*. *2023.07.21.550097. 10.1172/jci.insight.177002 PMC11382884 38713518

[77] Chen J, Zhang X, Li L, Ma X, Yang C, Liu Z, Li C, Fernandez-Cabezudo MJ, Al-Ramadi BK, Wu C, et al. Farnesyl pyrophosphate is a new danger signal inducing acute cell death. PLoS Biol. 2021;19(4):e3001134. 33901180 10.1371/journal.pbio.3001134 PMC8075202

[78] Jones AC, Pinki F, Stewart GS, Costello DA. Inhibition of Urea transporter (UT)-B modulates LPS-Induced inflammatory responses in BV2 microglia and N2a neuroblastoma cells. Neurochem Res. 2021;46(6):1322–9. 33675462 10.1007/s11064-021-03283-4

[79] Yu H, Meng Y, Wang LS, Jin X, Gao LF, Zhou L, Ji K, Li Y, Zhao LJ, Chen GQ, et al. Differential protein expression in heart in UT-B null mice with cardiac conduction defects. Proteomics. 2009;9(3):504–11. 19132680 10.1002/pmic.200701079

[80] Zhou L, Meng Y, Lei T, Zhao D, Su J, Zhao X, Yang B. UT-B-deficient mice develop renal dysfunction and structural damage. BMC Nephrol. 2012;13:6. 22289137 10.1186/1471-2369-13-6 PMC3293738

